# How do humans learn about the reliability of automation?

**DOI:** 10.1186/s41235-024-00533-1

**Published:** 2024-02-16

**Authors:** Luke Strickland, Simon Farrell, Micah K. Wilson, Jack Hutchinson, Shayne Loft

**Affiliations:** 1 The Future of Work Institute, Curtin University, 78 Murray Street, Perth, 6000 Australia; 2 The School of Psychological Science, The University of Western Australia, Crawley, Perth, Australia

**Keywords:** Human-automation teaming, Automation reliability, Cognitive model, Learning

## Abstract

In a range of settings, human operators make decisions with the assistance of automation, the reliability of which can vary depending upon context. Currently, the processes by which humans track the level of reliability of automation are unclear. In the current study, we test cognitive models of learning that could potentially explain how humans track automation reliability. We fitted several alternative cognitive models to a series of participants’ judgements of automation reliability observed in a maritime classification task in which participants were provided with automated advice. We examined three experiments including eight between-subjects conditions and 240 participants in total. Our results favoured a two-kernel delta-rule model of learning, which specifies that humans learn by prediction error, and respond according to a learning rate that is sensitive to environmental volatility. However, we found substantial heterogeneity in learning processes across participants. These outcomes speak to the learning processes underlying how humans estimate automation reliability and thus have implications for practice.

**Supplementary Information:**

The online version contains supplementary material available at 10.1186/s41235-024-00533-1.

## Introduction

In many human-automation teaming (HAT) contexts, a human operator supervises, verifies, or enacts advice from an automated system. For example, in the context of maritime surveillance, automation may monitor vessels for suspicious behaviour, and alert an operator to potential hostile targets. Automated systems are not perfectly reliable, and therefore a key variable determining HAT outcomes is the operator’s judgement of the reliability of the automation (i.e., of the probability that automation is correct). Operators are more likely to accept automated advice that they judge to be more reliable (e.g., Madhavan & Wiegmann, 2007; Rovira et al., 2007; Strickland et al., 2023), and to offload cognitive work to the advice (Wickens & Dixon, 2007). 

Research has shown that humans adjust their judgements of automation reliability based on task experience (e.g., Barg-Walkow & Rogers, 2016; Pop et al., 2015; Wiegmann et al., 2001). However, unsurprisingly, human judgements of automation reliability do not immediately adapt to match the “true” reliability (Hutchinson et al., 2022a, 2022b). It can be challenging to accurately infer the “true” level of reliability from operational experiences with the automation, particularly given the level of reliability may change (e.g., due to shifts in context). Changes in automation reliability can arise in field settings due to a range of factors including changes in environmental context, sensor noise, and adversarial attacks (e.g., cyber). For example, changing weather conditions can affect the reliability of air traffic control conflict resolution systems (Wang et al., 2022a, 2022b). Thus, learning about automation reliability embodies a general problem: learning from experience about a dynamic latent variable that is subject to shifts in its true state (Ez-zizi et al., 2023). 

The cognitive science literature contains multiple alternative models of learning that may provide insights into how humans learn about automation reliability. Broadly speaking, these models make predictions about precisely how learning unfolds after each experience. For the purposes of learning automation reliability, each experience involves observing whether an automation decision was correct. In addition to being theoretically informative, such models could potentially inform tools that predict other workplace phenomena (in addition to judgments of automation reliability), including the level of trust in and reliance on automation, variations in operator workload related to automation-use, and automation misuse/disuse rates (Lee & See, 2004; National Academies of Sciences & Medicine, 2022; Parasuraman & Manzey, 2010). Increasingly, formal models are being applied to the learning of automation reliability or related constructs (e.g., Chong et al., 2022; Hu et al., 2019; Wang et al., 2018). However, a limited number of learning processes have been explored, and there is little information about the relative performance of different models. Thus, the literature could benefit from a systematic evaluation of the viability of alternative models of the learning of automation reliability. To address this, the current study compares the relative performance of a set of alternative cognitive models of learning, using data from three previous experiments that measured judgements of automation reliability after each experience with automation (Hutchinson et al., 2022a, 2022b). 

### How might automation reliability be learned?

Much of the HAT literature focuses on *trust* in automation, the underlying cognitive state or attitude determining an individual’s willingness to rely on automation when uncertain (Hoff & Bashir, 2015; Lee & See, 2004). Trust in automation is a broad construct. Literature reviews and meta-analyses (Hoff & Bashir, 2015; Schaefer et al., 2016) have identified multiple factors contributing toward HAT trust, including dispositional factors (e.g., personality, expertise, age), situational factors (e.g., workload, competing demands, decision risk), and most crucially automation factors (reliability, transparency, anthropomorphism). Judgements of automation reliability, that is underlying beliefs of automation accuracy, are considered a particularly important subcomponent of trust (e.g., Schaefer et al., 2016). The more reliable the automation is judged to be, the more it makes sense to trust the automation. This paper focuses on identifying the mechanisms by which judgements of automation reliability are adapted based on task experience. 

Recently, Hutchinson et al. (2022a, 2022b) examined how judgements of automation reliability changed on an experience-by-experience basis (i.e., after observing each automation decision and whether it was correct). They reported a series of experiments in which participants provided reliability estimates after each automation experience. The participants experienced shifts in the automation’s reliability, but they were not informed when such shifts occur and hence could only infer them from experience. Hutchinson et al. (2022a, 2022b) found that on average, judgements of automation reliability did track towards true automation reliability, but lagged true reliability and did not fully “converge” over the course of experience using automation. Importantly, and consistent with a range of previous cognitive science literature, they found a “recency effect” (Jones & Sieck, 2003; Ludwig et al., 2012; Speekenbrink & Shanks, 2010), whereby the most recent performance of the automation had a large effect on judgements of reliability. The Hutchinson et al. (2022a, 2022b) studies were sensitive to this effect because judgements of reliability were measured after each experience with automation, rather than after blocks of multiple automation experiences as had been the case with most previous studies. Although Hutchinson et al. (2022a, 2022b) provided a more fine-grained analysis of judgements of automation reliability than previous work, they applied mixed-effects models to understand the effects of factors in the experiment, rather than models that speak directly to underlying cognitive processes. 

Computational cognitive models are powerful tools for understanding workplace performance (Boag et al., 2022; Byrne & Pew, 2009; Wu & Liu, 2021) that can provide insights into human adaptations to automation reliability. Crucially, cognitive models allow researchers to specify and test how cognitive processes describe the data of individuals. This is important when examining learning, where conclusions based on averaged models can be misleading when learning processes differ across individuals (e.g., Heathcote et al., 2000). 

Wang et al. (2018) provided a model of how judgements of automation reliability and trust in automation evolve on an experience-by-experience basis. They applied a Bayesian model that assumed a single “true” latent reliability level, and that individuals learned a belief distribution of that reliability according to a beta-binomial model, with the reliability determined by the mean of that distribution. More recent work has demonstrated the potential of this approach to predict operator trust in automation in “real time” (Guo et al., 2020). Such real time predictions could potentially inform adaptive automation (e.g., that determines and signals when operator trust may be too high or too low; Feigh et al., 2012; Griffiths et al., 2023). However, although Wang et al. (2018) found that their Bayesian model provided a reasonable account of some participants’ reliability judgements, other participants’ judgements were not well accounted for due to faster shifts in learning than predicted. Notably, because the model assumes that all previous experiences with automation are exchangeable (i.e., more recent experiences are weighted equally to less recent experiences), it cannot account for recency effects. This model assumption conflicts with the strong and consistent recency effects on judgements of automation reliability observed by Hutchinson et al. (2022a, 2022b). 

A range of cognitive models of learning, particularly probability learning, might offer alternative accounts of how human operators learn about automation reliability. In probability learning people must learn about the probability of a response being correct or being rewarded, and a major interest has been in how people track fluctuations or switches in that probability over time. By considering learning about automation reliability as a probability learning challenge, we can consider how probability learning models apply to automation reliability, and the results of Hutchinson et al. (2022a, 2022b) in particular. The influential “delta rule” model assumes that participants learn according to prediction error after every experience (Lee et al., 2020). Under this model, the current estimate of automation reliability can be thought of as a recency-weighted average of previous automation experiences (Sutton & Barto, 2018). The delta rule’s applicability to learning of probabilities has been criticized because it does not capture the stepwise way that participant probability judgements can shift in some experiments (e.g., Gallistel et al., 2014). However, some recent research suggests that stepwise shifts in probability judgements may be an artefact of design choices in previous studies, such as asymmetry in the effort required for participants to update versus not update probability judgements (Forsgren et al., 2023), rather than a reflection of the true functional form of latent probability estimates. 

In some settings, it is important to adjust learning rates based upon environmental volatility (McGuire et al., 2014; Nassar et al., 2010). When the environment has recently shifted, old observations are less relevant to the current probability estimate, and hence their influence should be diminished with a high learning rate. Although variable learning rate delta-rule models can be demanding to estimate, one tractable approach is to apply a “two-kernel” delta rule that runs two concurrent delta-rule learning processes—one fast learner and one slow learner—and use the estimates from the slower learner by default but switch to estimates from the faster learner when prediction error is sufficiently high (Forsgren et al., 2023; Gallistel et al., 2014). Indeed, it appears that a two-kernel delta rule provides a better account of probability estimates than a standard delta rule (Forsgren et al., 2023). 

Previous work suggests promise for delta-rule approaches in describing HAT outcomes. Hu et al. (2019) tested a model of trust dynamics that included a delta-rule learning component as well as additional updating terms based on cumulative experience, bias, and relative weightings for different types of automation failures. This model was shown to describe an accurate account of trust in automation, where trust was operationalized as the (group-averaged) probability of choosing to trust (from a binary trust/distrust response). Further, Chong et al. (2022) found the same model to provide an accurate account of grouped trust in automation ratings, and extended the approach to account for how self-confidence (i.e., trust in one’s own judgements) changes with experience. The Hu et al. model is a model of grouped data that learns from error rates and reported trust aggregated across participants on each trial. We focus on simpler delta-rule models that directly model how people learn from individual trial events, and can be fit to individual data. 

An alternative to delta-rule models that may be applicable to automation reliability is Gallistel et al.’s (2014) model of perceived probability. This model, which was designed in part to account for stepwise shifts in probability judgements, predicts that judgements of probability are only adapted occasionally when participants’ belief that they are incorrect reaches a threshold. In that event, a sudden and potentially large shift in probability judgements can occur. Gallistel et al.’s model behaves this way by implementing a hypothesis test, in which participants test whether their current judgement of probability is “broken” before deciding whether to adjust it. It embodies the principle that “if it ain’t broke, don’t fix it” (Gallistel et al., 2014), and thus, we refer to it henceforth as the “if it ain’t broke” (IIAB) model. 

Learning of automation reliability could also be described by memory sampling models. Memory sampling can take various forms. For example, recency-based memory sampling could sample previous experiences with probabilities proportionate to delta-rule weights (Bornstein et al., 2017). This model would make similar predictions to the delta-rule model if many memory samples were concurrently recalled and averaged to form a judgement. However, if only a small number of previous experiences are sampled (e.g., one previous experience) predictions can be quite different to the delta-rule model (Bornstein et al., 2017). Alternatively, memory sampling could take a variety of other forms. For example, participants might probabilistically either remember their most recent experience, or rely on a process that approximates the average of all previous experiences. 

A final mechanism considered here is the “contingent sampling” discussed by Hochman and Erev (2013). In their model, previous experiences only inform the current estimate if they were preceded by sequences matching small samples of recent experience. For example, if a participant’s last two experiences involved automation being correct (two decisions back) and then incorrect (one back), then the reliability of automation for the current decision could be estimated using previous occasions which were preceded by the same sequence of automation accuracies (correct and then incorrect). If after previous instances of this sequence (correct, incorrect) the automation was subsequently correct, then the participant would estimate a high reliability when encountering the sequence again. 

## The current study

We aimed to formally compare cognitive models of how learning affects judgements of automation reliability over time. We did so by re-evaluating the Hutchinson et al. (2022a, 2022b) judgements of automation reliability data using alternative cognitive models of learning, with each fitted to the time series of automation reliability judgements of each individual participant. In these studies, participants performed a maritime vessel classification task with the assistance of an automated decision aid. This task is broadly representative of modern work domains in which individuals must monitor displays to classify or make other decisions about representations of real-world objects. After each automation experience, participants were asked about their judgement of the automation’s future reliability. The true reliability of the automation varied across the three experiments (representing eight between-subjects conditions). Furthermore, there were points at which automation reliability shifted within each condition, with the nature and timing of the shifts differing across conditions. Participants were not warned of these shifts in reliability, and thus could only learn about them from experience. Applying learning models to a range of different experimental conditions provides an opportunity to test the generality of their assumptions.

Previous studies examining learning of automation reliability/trust have largely focused on the viability of a single approach to modelling learning (e.g., Hu et al., 2019; Wang et al., 2018). In contrast, our aim is to compare the relative utility of a range of cognitive models to explain the learning of automation reliability in individuals. To do so, we compare a set of models using the Hutchinson et al. (2022a, 2022b) judgements of automation reliability data. This includes a Bayesian model similar to that specified by Wang et al. (2018); the delta-rule learning model; a two-kernel variant of the delta-rule model; three memory sampling models; and the IIAB model. We apply a formal model comparison approach (Myung & Pitt, 1997) to test, at both the group level and the level of individual participants, which learning processes best explained participant judgements of automation reliability.

## Hutchinson et al. (2022a, 2022b) experiments

### Participants

As reported by Hutchinson et al. (2022a, 2022b), participants were 260 undergraduate students from the University of Western Australia who received course credit. Twenty participants were excluded because we suspected they were not engaged with the reliability judgement task: either because visual inspection of the time series of judgements revealed runs of many 0%, 50%, or 100% judgements (as originally identified by Hutchinson et al., 2022a, 2022b), or because they made the default response of 50% reliability more than half the time overall. All studies received approval from the University of Western Australia’s Human Research Ethics Office. 

### Maritime vessel classification task

**Fig. 1:**
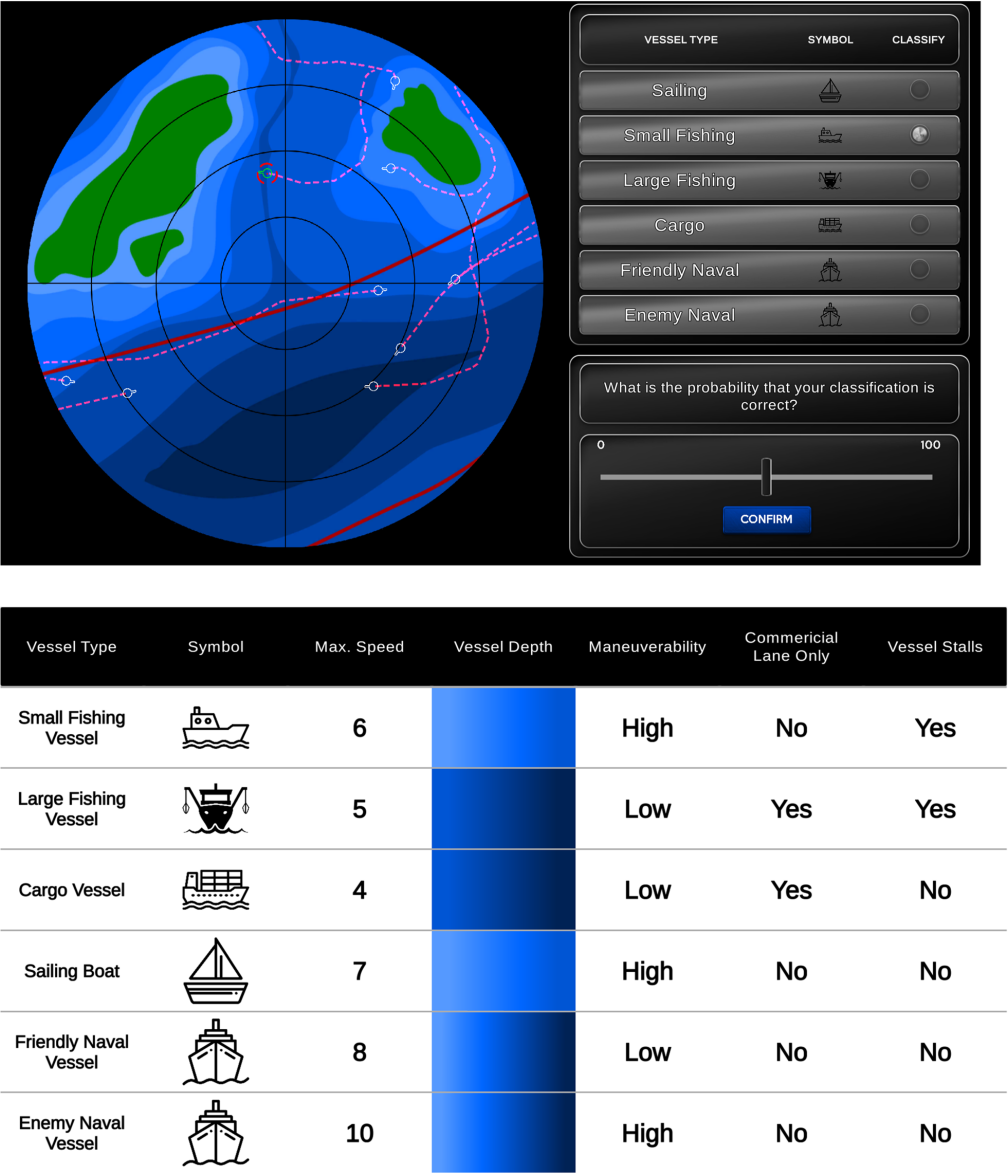
Screenshots of the maritime vessel classification task. Figure adapted from Hutchinson et al. (2022a). *Note* The top panel is a screenshot of the task presented on the participants’ primary (left) monitor, and the bottom panel of the display on their secondary (right) monitor

Detailed descriptions of the task are available in Hutchinson et al. (2022a, 2022b). Contacts were represented by small white circles appearing within the blue areas of a bathymetric display (Fig. 1). At the start of each trial, a specific contact was highlighted and participants were required to classify it using six possible classifications (e.g., cargo vessel). The classification was based on several rules provided in Fig. 1. After classification, participants were asked to provide a judgement of their choice’s reliability (their confidence). Subsequently, participants were presented with a classification recommendation from the automated advice and given an opportunity to accept or reject it. Finally, they were provided feedback on the correct decision, their own decision, and the automated advice. On the same screen, they were asked to judge the automation’s future reliability with a slider. Specifically, they were asked: “*what is the probability that the automation’s next classification will be correct?*”. They submitted this judgement on a percentage scale ranging from 0 to 100 (the default response was 50). This process was repeated for each contact required to be classified.

### Experiment designs

Each experiment included the same vessel classification task, but the experiments differed in numbers of contacts and the levels of automation reliability presented throughout. Experiment 1 included 15 trials, with eight contacts to classify in each trial. There were two between-subjects conditions. In the “high reliability” condition, in the first five trials (40 contact classifications) the automation was 90% accurate. Specifically, in one trial the automation was correct 8/8 times and in the four other trials it was correct 7/8 times. In the “low reliability” condition, the automation was 60% accurate for the first five trials. Specifically, in one trial automation was correct 4/8 times, and in the four other trials it was correct 5/8 times. From trials 6–15 (decisions 41–120), automation reliability in both conditions was 75%, correct 6/8 times for each trial. Participants were not informed about the change in automation reliability.

Experiments 2 and 3, which examined the effect of more frequent and large changes in reliability, included 16 trials each, with 10 contacts on each trial. In Experiment 2, there were three conditions. In the simplest condition, the “Constant Reliability” condition, automation reliability was 75% throughout. Specifically, automation was correct 7/10 times for half the trials, and 8/10 times for the other half. In the other two conditions, reliability could be classified according to four sets of 4-trial (40 contact classification) long phases. In the “Start-High” condition, the automation was 95% reliable for the first phase. Specifically, automation was correct for 10/10 times for half the trials, and 9/10 times for the other half. In the “Start-Low” condition, the automation was 55% reliable in the first phase. Specifically, automation was correct for 5/10 times for half the trials, and 6/10 times for the other half. At the end of each 4-trial phase, the reliabilities then switched across the Start-high and Start-low conditions. For example, in the second phase, reliability was 55% for the Start-High condition and 95% for the start-low condition. 

In Experiment 3, there were three conditions, each with 16 total trials that contained 10 contacts each. In each condition, automation reliability was initially 90% for the first 4 trials and returned to 90% for the last 8 trials. Specifically, automation was correct 9/10 times on each trial. However, there was a “drop” in reliability for trials 5–8, and the size of this drop depended upon the condition. In the “large drop” condition, automation reliability dropped down to 30% (automation correct 3/10 times per trial), 50% (automation correct 5/10 times per trial) in the “Medium Drop” condition, and in the “Small Drop” condition automation reliability dropped to 70% (automation correct 7/10 times per trial). 

**Fig. 2:**
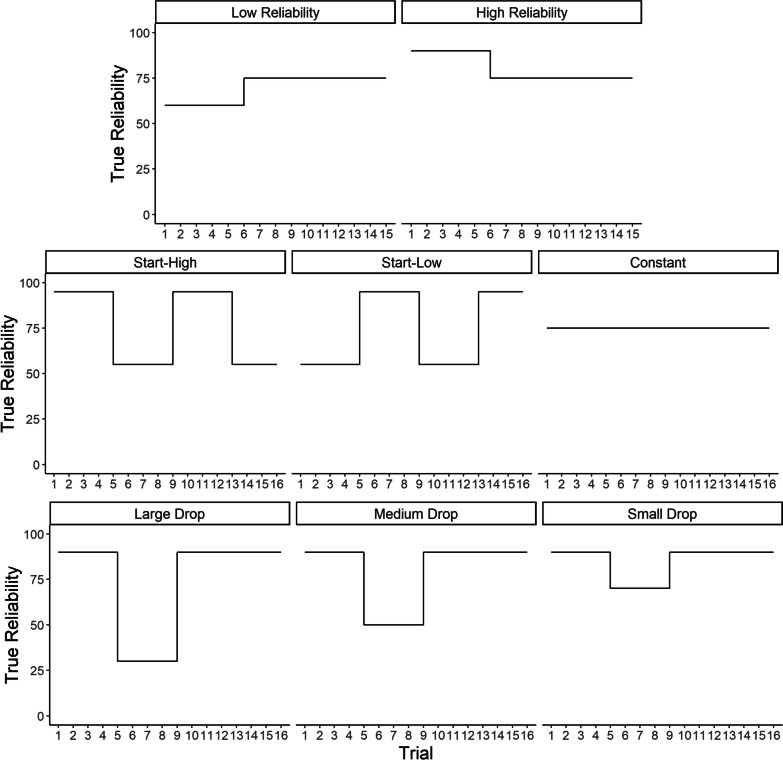
Visualization of the true reliability of automation in each experiment of Hutchinson et al. (2022a, 2022b). *Note* Rows correspond to experiments and panels experimental conditions. Lines follow a stairstep pattern, shifting exactly for the blocks where true reliability differed from immediately preceding blocks. For example, in the low reliability group, true reliability changed to 75% at the beginning of block 6, and thus the stairstep moves vertically up from 60 to 75% in block 6

Participants were not instructed about the level of automation reliability, except in Experiment 3. In that study, they were initially instructed that the historical performance of the automation indicated 90% reliability. A visualization of switches in reliabilities across each experimental condition is shown in Fig. 2.

## Cognitive modelling

All analyses and simulations were conducted using the R programming language (R Core Team, 2022). We created a binary variable indexing whether the automation was correct—1 for correct and 0 for incorrect—and the time series of this variable was the input to each learning model. To match the scale of automation correctness (0–1), reliability judgements were divided by 100. To define model likelihoods, we assumed that observed judgements of automation reliability were drawn from a truncated normal distribution (implemented in the 'truncnorm' package; Mersmann et al., 2018), bounded between 0 and 1. The latent mean of this distribution was determined by the learning process, and a standard deviation parameter, *σ*, was estimated for each model. Thus, the latent mean in the model represents the belief according to the learning process, and the *σ* parameter represents noise independent of the learning process. All model parameters were estimated for each individual participant using a maximum likelihood approach. We describe model-fitting details in Additional file 1.

In the following, we first introduce the implemented cognitive models of learning, and then compare relative (group-level) model performance for each experimental condition. Subsequently, we examine the absolute performance (fit) of the most favoured model, the two-kernel delta rule, and report its estimated parameters. We conclude by exploring individual differences (heterogeneity in learning processes). 

### Learning processes

#### Static-environment Bayesian model

Following Wang et al. (2018), we fit a Bayesian model that assumed there was an unchanging single “true” state of automation reliability. Belief about automation reliability was characterized by a beta distribution:

1 $$r_{t} \sim {\text{Beta}} \left( {p + N_{{{\text{AC}}}} , q + N_{{{\text{AI}}}} } \right).$$

$N_{{{\text{AC}}}}$ and $N_{{{\text{AI}}}}$ indicate the number of total times automation was correct and incorrect. The parameters $p$ and $q$ determine the participants’ prior belief about automation reliability. The posterior mean of $r_{t}$ determined the latent mean reliability judgement.^1^

#### Delta-rule models

We implemented a typical delta-rule learning model (Lee et al., 2020), in which participants updated reliability perceptions based upon prediction error from the most recent automation experience:

2 $$r_{t} = r_{t - 1} + \alpha \left( {{\text{AC}}_{t} - r_{t - 1} } \right)$$

where AC stands for automation correctness (1 for automated advice was correct, 0 for incorrect). This learning process introduces two parameters: the initial estimate of automation reliability $r_{0}$, and the learning rate $\alpha$, which controls the rate at which learning occurs. The delta rule can also be considered a recency-based weighted average of previous experiences with automation (Sutton & Barto, 2018), of the form:

3 $$r_{t} = (1 - \alpha )^{t } r_{0} + \mathop \sum \limits_{i = 0}^{t - 1} \alpha (1 - \alpha )^{i} {\text{AC}}_{t - i} .$$

People may track or adapt to sharp changes in states, such as those implemented in our studies, with variable learning rates (McGuire et al., 2014). To test such a mechanism in a tractable way, we implemented a “two-kernel” delta rule (Forsgren et al., 2022; Gallistel et al., 2014). This learning model assumes that participants simultaneously track two estimates using delta-rule learning as formalised in Eq. 2, with each delta learner sharing the same start point but having separate learning rates. When estimates from the slower delta learner are substantially different to estimates from the faster delta learner (as determined by a threshold parameter), this signals environmental volatility. In such cases, the estimate from the faster delta learner is used to provide a response. When the difference between fast and slower delta learners is smaller than the threshold, the estimate from the slower learner is used. The two-kernel delta rule incorporates four parameters: the initial reliability estimate $r_{0}$, the slower learning rate *α*_slow_, the faster learning rate *α*_fast_, and the threshold (*T*) determining use of the estimate from the fast learner.

#### Memory sampling models

Three memory sampling models were tested. In the first memory sampling model (Bornstein et al., 2017), either one previous automation experience or the initial belief (prior to any experience) about automation reliability is sampled from memory for each judgement, and the sampled memory determines the reliability estimate. Sampling probabilities are determined by the same recency form as weights are in the delta rule (Eq. 2), controlled by a parameter α_sampling_ analogous to the learning rate. There was also an initial state parameter *r*0_sampling_recency_, with similar considerations to $r_{0}$ from the delta-rule model. 

The second memory sampling model assumed that either the most recent experience was sampled (with probability determined by a parameter prob_*t*_), or the average of all previous automation experiences (with probability 1 − prob_*t*_). Whichever is sampled determines the reliability estimate. This sampling model also has two additional parameters determining the initial (pre-experimental) perception of automation reliability (*r0*_sampling_first_average_) and the weight controlling its contribution to the aforementioned average of previous automation experiences (weight_*r0*_). 

The third memory sampling model relied on contingent sampling (Hochman & Erev, 2013). The premise of this model is that participants track some small sample (determined by a discrete parameter *m*) of recent events (e.g., two events: automation was correct for the last contact, and incorrect for the contact before), remember previous instances with identical recent event histories, and use the outcomes that previously followed those identical histories to predict what will happen for the current sequence. Predicted reliability was based upon previous memory samples that matched the most recent *m* automation experiences. For example, for *m* = 2, for a situation where automation had been correct about the previous last two contacts, then the current estimate of reliability would be determined by the average reliability following previous instances where the automation had been correct for the two contacts prior. Following this example, imagine that automation had been correct on the previous two contacts. If there had been two previous such sequences, one followed by correct automated advice, and the other followed by incorrect automated advice, then the current reliability estimate would be 50%. In cases where no previous contact histories matched the most recent contacts, a mismatching contact history of length *m* was randomly sampled from the histories that had been observed. For the initial judgements where less than *m* previous contacts had been observed, the reliability estimate was given by a parameter *r0*_sampling_contingent_. 

#### IIAB model

We implemented the IIAB model of Gallistel et al. (2014). In this model, participants track shifts in probability discretely based on the observations since perceived “change points”. The initial belief about automation reliability is characterized by (the mean of) a beta distribution with prior parameters *p* and *q*. At specific change points the information preceding the change point is effectively ignored, and information since the last change point is used to estimate the current probability. This leaves the challenge of deciding that a change has occurred.

To detect changes, judgements begin with a test of whether evidence against the null hypothesis (i.e., the hypothesis that the estimate is not “broken”) exceeds some threshold level (*T*_1_). If so, the model enters a second stage which tests whether to modify the record of tracked change points. A Bayesian test is performed to determine whether there is sufficient evidence in favour of adding an additional change point (greater than some threshold parameter *T*_2_), and if so, a change point is added. If not, the model has a “second thought” about the last change point it added. Another Bayesian test is conducted, this time in favour of removing the last change point. If the evidence in favour of dropping the change point is greater than parameter *T*_2_, the change point is dropped. Reliability estimates are based on observations since the last change point. Specifically, a beta distribution is updated using the number of times the automation was correct versus incorrect since the last change point. The mean of this distribution is the reliability estimate. Similarly, a beta distribution reflecting the perceived probability of change points, with prior parameters $p_{{\text{change point}}}$ and $q_{{\text{change point}}}$, is updated according to the number of contacts for which there were change points versus contacts for which there were not change points during the experiment.

#### No updating (baseline) model

Finally, we considered a simple “no updating” model, where latent mean reliability estimates were simply given by an intercept parameter $r_{0}$. Although this model is unlikely to account for participants who are engaged in both learning automation reliability and reporting their judgements thereof, it is useful as a baseline. Specifically, any model that successfully describes how participants learned about automation reliability should fit better than this model.

Table 1 contains a list of each cognitive model considered, a brief description, and parameters for each model. Additional file 1 includes plots visualizing the predictions of each alternative learning model.

**Table 1:** A list of the learning models fitted to judgments of automation reliability, and associated learning parameters

Model	Description	Learning parameters
Bayesian	Bayesian learning of automation reliability assuming a single true state	$p, q$
Delta	Judgements of automation reliability are updated based upon the prediction error (delta) between the previous reliability estimate and the current automation accuracy	$r_{0}$, $\alpha$
Two-Kernel Delta	Two simultaneous delta-rule learners track automation reliability. Estimates are taken from the slower learner unless the difference between the two processes is above a threshold, signalling a shift in the environment, in which case the fast delta learner is used	$r_{0}$, *α*_fast,_ *α*_slow_, *T*
Sampling (proportional to delta weights)	A single previous memory is sampled to inform the current estimate of automation reliability. Previous experiences are sampled proportionately to their weights under a delta-rule updating process	*r*0_sampling_recency_, *α*_sampling_
Sampling (last/average)	Samples either the most recent experience with automation, or the average reliability of all previous experiences	*r*0_sampling_last_average_, weight_*r*0_, prob_*t*_
Contingent Sampling	Automation reliability is assumed to be sensitive to the history of automation accuracy over the recent *m* contacts. Thus, the reliability estimate is based on previous cases where the history of automation accuracy *m* contacts back matches the history *m* contacts back in the current instance	*r*0_sampling_contingent, m_
IIAB	Estimates of automation reliability are updated in a stepwise manner when a “change point” is identified. Sometimes, the model has “second thoughts” and expunges or updates a previous change point	*T*_1_, *T*_2_, $p, q, p_{{\text{change point}}} , q_{{\text{change point}}}$
No updating	No learning process	$r_{0}$

See text for more in-depth descriptions. Note that fitting each model also involved estimating a latent standard deviation parameter, *σ*, indexing noise in responding that is independent of the learning process

### Model comparison

We calculated the Bayesian information criterion (BIC) to evaluate model performance for each participant (Myung & Pitt, 1997). Differences in BIC can be used to compare the relative predictive performance of models, taking consideration of both their fit and parsimony (number of parameters). A larger BIC indicates less support for a model (due to poorer fit, more model complexity, or both). To evaluate overall results for each experimental condition, we summed BICs across participants. Results are summarized in Table 2. Overall, our findings favour the two-kernel delta-rule model, with it being the most supported model by summed BIC for seven of eight Hutchinson et al. (2022a, 2022b) experimental conditions, and second most supported in the remaining condition. The delta-rule model also performed quite well, being the second most supported model by BIC in five conditions, the third most supported in two conditions and the fourth most supported in one condition. Interestingly, the memory sampling model that sampled proportionately to delta weights was most supported by summed BIC in Experiment 2’s start-low condition, and second-most supported in the Constant condition. However, follow-up analysis of the start-low condition (Additional file 1) revealed that this summed BIC preference was strongly influenced by a single participant who responded in an idiosyncratic way. Excluding that participant resulted in the two-kernel delta rule being favoured for the start-low condition.

**Table 2:** Group BIC values for each model for each experimental condition

Model	One		Two			Three		
	High	Low	Start-high	Start-low	Constant	Large drop	Medium drop	Small drop
*Experiment*								
Two-kernel Delta	0	0	0	515	0	0	0	0
Delta	342	226	449	728	387	335	375	87
Sampling (proportional to delta weights)	990	1147	2171	0	219	1549	1950	97
Sampling (last/average)	679	728	1790	931	318	1542	1548	171
IIAB	1267	982	346	1436	1416	809	1013	502
Bayesian	1016	873	2089	1956	1122	2645	1764	376
Contingent Sampling	4034	5249	3695	3942	4648	3527	4218	3175
No updating	1214	1124	2276	2023	981	2978	2100	274

We report BIC values after subtracting the BIC for the most supported model for each experiment condition (Hence, the most supported model for each experimental condition has a value of 0). We report BIC in this manner because it is the differences between BICs that matter for the purposes of model comparison (Kass & Raftery, 1995) and it is easier to see which model is best fitting for each condition, and the relative performance of other models to that best fitting model

### Model fit

**Fig. 3:**
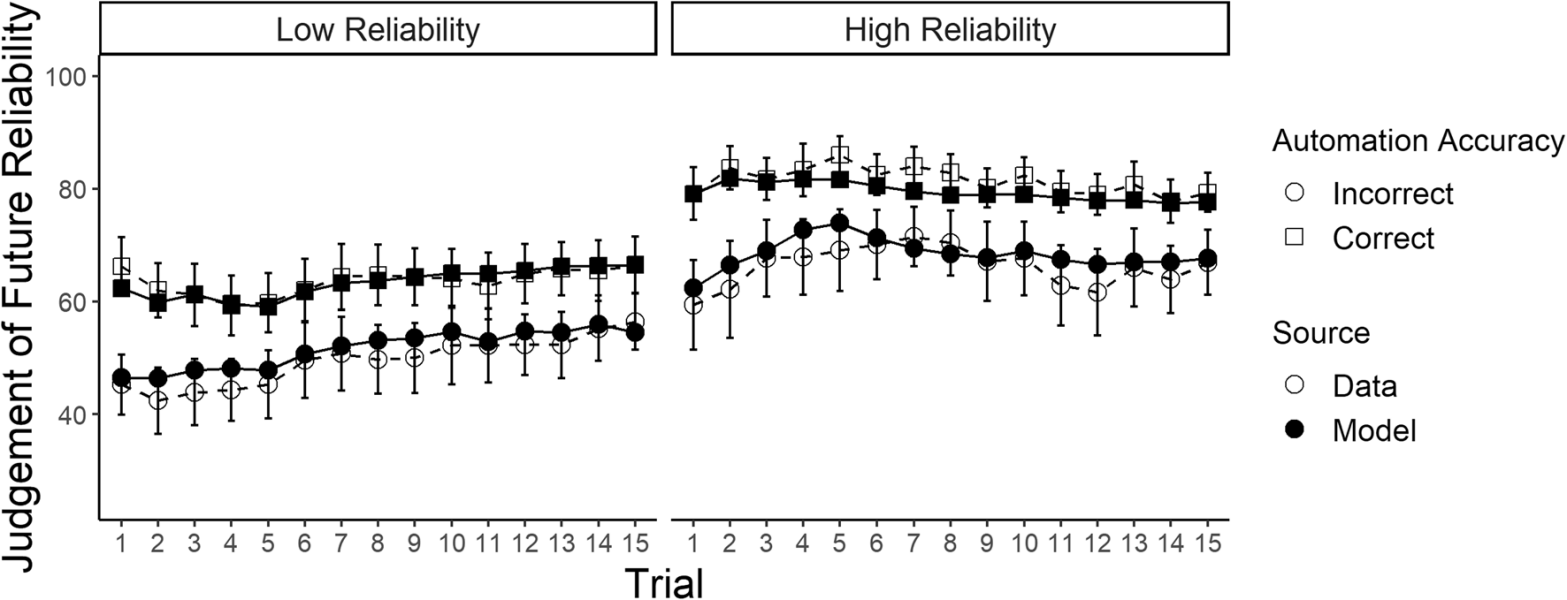
Averaged predictions of the two-kernel delta-rule model for experiment 1 (Hutchinson et al., 2022a). *Note* The data correspond to the white circles, the model mean predictions to the black dots. The error bars display the data means plus or minus the standard error.

**Fig. 4:**
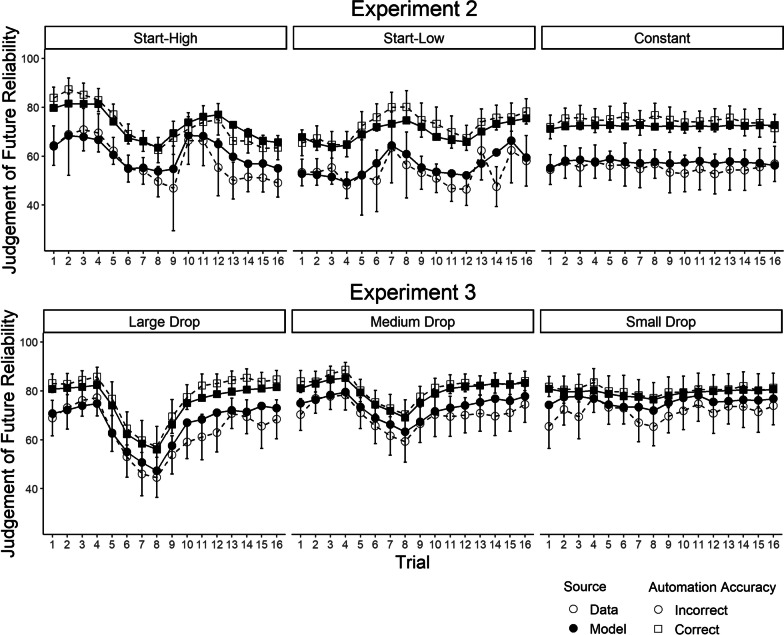
Averaged predictions of the two kernel delta-rule model for experiment 2 and 3 (Hutchinson et al., 2022b). *Note* The data correspond to the white circles, the model mean predictions to the black dots. The error bars display the data means plus or minus the standard error

Although our results indicate that the two-kernel delta rule was overall the best fitting of the models considered, a remaining question is whether it was a reasonable description of the data in absolute terms. To evaluate this, we plot average model fit across the experiments in Figs. 3 and 4. To summarize long-running effects, we examine model fit across “trials” (i.e., small blocks of decisions about contacts). Given the importance of the effect of the most recent automation advice (Hutchinson et al., 2022a, 2022b), we separately average and plot judgements after participants had just received accurate automation advice, and judgements after participants had just received inaccurate advice. Overall, these figures demonstrate that the two-kernel delta-rule model provided a reasonable “absolute” fit to patterns in judgements of automation reliability across all three experiments. The model also provided a reasonable fit to intra-trial variability in reliability judgements, as well as the discrepancy between participant reliability judgements and the empirically observed reliabilities within each trial, both of which are plotted in Additional file 1. Plots of fit for the alternative models can also be found in Additional file 1.

In Additional file 1, we detail *why* the two-kernel delta-rule model provided superior fits to the simpler, standard one-process delta-rule model. Our findings indicated that although fits of the single-process delta rule were reasonably good, it struggled to simultaneously capture both the effects of the most recent automation accuracy and the longer-running effects of true automation reliability (i.e., effects of the true automation reliability state on series of reliability judgements across trials). Specifically, we found the estimated single-process delta rule model underpredicted the effects of the most recent automation accuracy on judgements, and that forcing the learning rate to be more in line with the observed effect of the most recent automation accuracy resulted in misfit of longer-term learning associated with the true automation reliability state. In contrast, the two-kernel delta rule was able to produce strong effects of recent automation experience by sometimes switching to the “fast” learning process for some judgements, but with the flexibility to switch back to the “slow” learning process for other judgements, allowing better fit to long-running learning effects. This nuanced distinction between model predictions would have been difficult to discern in the absence of formal modelling. 

### Model parameters

**Fig. 5:**
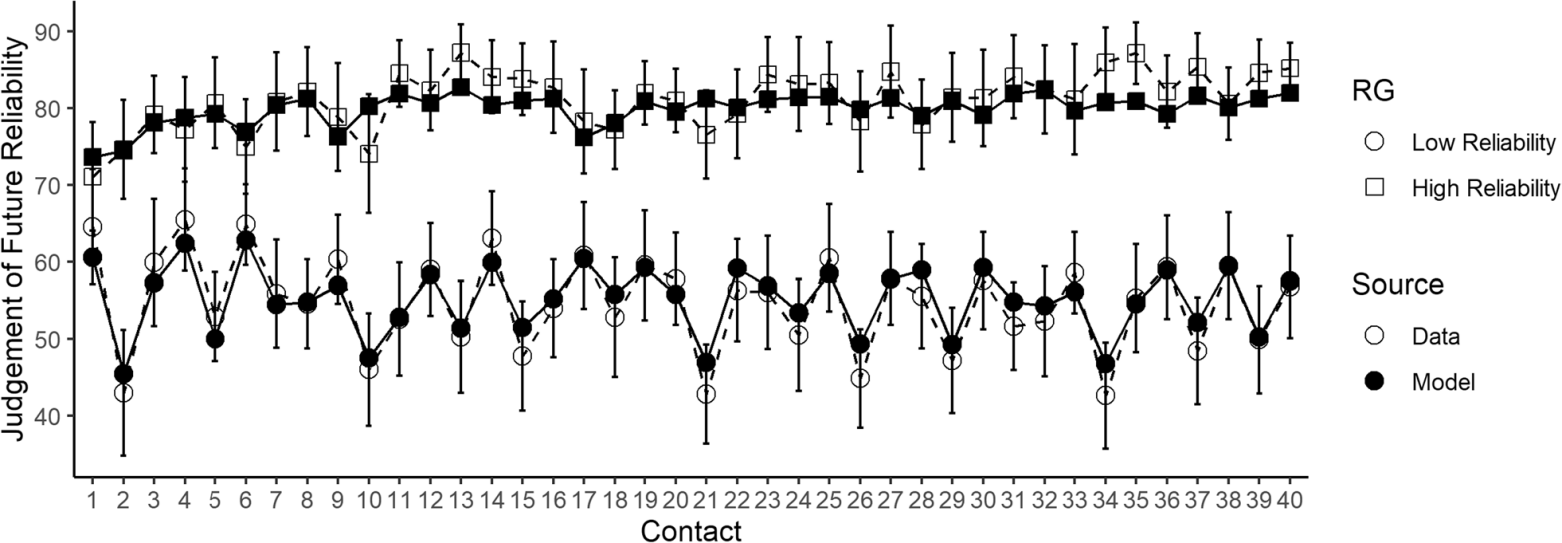
Model fits of the two-kernel delta rule broken down by individual contacts, for the first 40 contacts. *Note* The data correspond to the white circles, the model mean predictions to the black dots. The error bars display the data means plus or minus the standard error. Shapes indicate the experimental condition (reliability group; RG)

The estimated parameters of the two-kernel delta-rule model are in Table 3. Parameters of the other alternative models are available in Additional file 1. We tested differences in model parameters across conditions in each experiment (see Additional file 1 for tests). Generally, parameters did not vary substantially across conditions, but there were two exceptions. First, in Experiment 1 the $r_{0}$ parameter was higher in the high reliability condition than the low reliability condition. Plotting the model fits to judgements after each individual contact (Fig. 5) suggested this was associated with the model slightly over-estimating differences in group-averaged reliability perceptions on the very first contact. Second, in Experiment Three the *σ* parameter was larger in the large drop condition than in the Medium Drop condition, which was similar to *σ* in the Small Drop Condition. This suggests that the large drop condition may have induced extra variability in perceptions that was not fully accounted for by the two-kernel delta-rule learning model.

**Table 3:** Estimated parameter values of the two kernel delta-rule model, presented as M (SE)

Experiment	Condition	$r_{0}$	*α*_slow_	*α*_fast_	*T*	*σ*
One	Low reliability	0.58 (0.05)	0.03 (0.01)	0.38 (0.05)	0.35 (0.05)	0.14 (0.01)
	High reliability	0.78 (0.05)	0.03 (0.01)	0.35 (0.06)	0.31 (0.05)	0.14 (0.01)
Two	Start-high	0.74 (0.06)	0.03 (0.01)	0.35 (0.06)	0.30 (0.05)	0.14 (0.01)
	Start-low	0.67 (0.05)	0.01 (0.004)	0.44 (0.07)	0.34 (0.06)	0.21 (0.03)
	Constant	0.69 (0.05)	0.01 (0.01)	0.44 (0.07)	0.31 (0.04)	0.16 (0.02)
Three	Large drop	0.84 (0.04)	0.04 (0.01)	0.31 (0.06)	0.29 (0.05)	0.14 (0.01)
	Medium drop	0.80 (0.04)	0.02 (0.01)	0.11 (0.04)	0.15 (0.04)	0.10 (0.01)
	Small drop	0.77 (0.06)	0.01 (0.01)	0.21 (0.06)	0.33 (0.07)	0.11 (0.01)

### Individual differences

To explore heterogeneity in learning processes across participants, we focused on participants whose data conclusively supported one model above all competitors. To evaluate this, we converted differences in BICs to an approximate Bayes Factor (Kass & Raftery, 1995). For participants where the Bayes Factor comparing the most supported model to the next best competitor was < 3.2, we categorized the result as inconclusive based on Jeffreys’ scale of evidence (Jeffreys, 1961). Overall, one of the tested models was conclusively favoured for 83% of participants. Table 4 presents the percentage breakdown of these participants, categorized by specific favoured model, for each condition and experiment.

**Table 4:** The proportion of participants for whom BIC conclusively favoured each model for each experimental condition

Model	One		Two			Three		
	High (%)	Low (%)	Start-high (%)	Start-low (%)	Constant (%)	Large drop (%)	Medium drop (%)	Small drop (%)
*Experiment*								
Two-kernel delta	42	37	37	22	26	17	48	21
Delta	15	15	11	17	9	22	13	5
Sampling (proportional to delta weights)	6	0	0	26	26	9	0	11
Sampling (last/average)	3	0	7	4	17	17	4	5
IIAB	9	19	37	13	4	17	9	11
Bayesian	3	0	0	0	4	9	0	0
Contingent Sampling	0	0	0	0	0	4	0	0
No updating	21	30	7	17	13	4	26	47

Presented as a percentage of the 83% of participants for which one model was conclusively favoured

There was substantial heterogeneity in the learning models favoured. Unsurprisingly (given the group results), the two-kernel delta rule and the delta rule were substantially represented across participants, with one of the two models being favoured for 26–61% of participants with unambiguous results, depending on condition. The recency-based memory sampling models were also represented across participants, being favoured for 0–43% of participants, depending upon condition. The IIAB model was also represented, best accounting for 9–37% of unambiguous participants, depending on condition. Notably, the no-learning model was favoured for 7–47% of unambiguous participants, suggesting that a proportion of participants in the study were best accounted for by a model without a learning process, particularly in the “Small Drop” condition in Experiment 3 where there was a relatively minor and transient reliability change. The Bayesian model accounted for only a small number of participants, 0–9%, depending on experimental condition; and the Contingent Sampling model was only the best model of 4% of unambiguous participants in the “large drop” condition, and none in other conditions.

### Exploring individual differences: stratified fits

Due to the heterogeneity in learning models supported across participants, we explored the data patterns to which different models provided the best fit. To do so, we examined fits of models for groups of participants, stratified by the model that was supported for that group of participants. For the sake of brevity, we focused on Experiment 2 and examined models that were supported for at least 20% of participants within the relevant condition. This cut-off was applied to avoid plotting noisy results, with the specific choice of 20% being somewhat arbitrary. We chose Experiment 2 because it included a baseline constant true reliability condition, and multiple switches in the true reliability of the other two conditions, which are both helpful features to illustrate the behaviour of alternative models. Further, it was the only experiment with a condition in which the two-kernel delta-rule model was not the most supported by summed BIC, and overall indicated more heterogeneity in the modelling results.

**Fig. 6:**
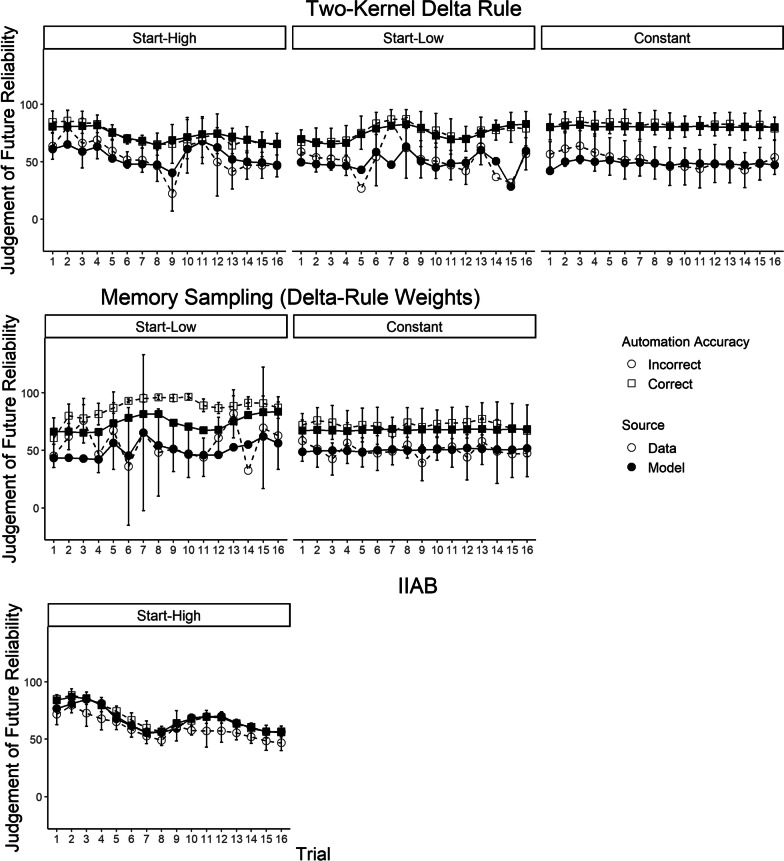
Experiment 2, model fits stratified by participants for the winning model. *Note* Panels for models/conditions are only included for conditions where more than 20% of participants within that condition were supported by the model. The data correspond to the white circles, the model mean predictions to the black dots. The error bars display the data means plus or minus the standard error. Note that in some cases, there were little data per participant and thus error bars could be large. Further, in some cases there were only data for one participant, in which case error bars are omitted

Figure 6 depicts stratified model fits to Experiment 2’s reliability judgements across participants, stratified by fits from the model that the BIC supported for their data. Participants best fitted by the two-kernel delta-rule model generally demonstrated a strong effect of the most recent automation experience on reliability judgements, and weaker but non-trivial long-running effects of true automation reliability on judgements. Participants best fitted by the memory sampling model (proportional to delta-rule weights) demonstrated a strong recency effect, but other patterns in their data were less clear. Notably, model fits to participants supported by the recency sampling model were not very visually compelling, suggesting there were trends in these participants' data that the model did not entirely explain, despite it being the “best” model of their data in terms of BIC relative to the other models. Participants best fitted by the IIAB model displayed little effect of the most recent automation experience, but moderate long-running learning effects.

### Exploring bias in probability estimation

The only source of bias considered in the presented models is that existing at the start of the experiment, and which is downweighted as learning progresses. However, there may be systematic and ongoing biases in the production of automation reliability judgements, consistent with findings that humans over- and under-estimate probabilities in other contexts such as decisions under risk (e.g., Tversky & Kahneman, 1992; Zhang & Maloney, 2012). Although we cannot be certain about the form of specific biases in this context, a broad class of the biases evident in human probability estimation can be captured by the “linear in log odds” (LLO) model (Zhang & Maloney, 2012).^2^ In this model, the log odds of the human probability estimate are linearly related to the log odds of the “true” probability. This model can produce a wide range of S- and inverted S-shaped functions describing biases in the production/estimates of probabilities. It includes two parameters controlling the shape of the probability bias function.

To test the robustness of our conclusions to potential distortions in estimated automation reliability, we augmented the models reported above in text to allow an LLO transformation of automation reliabilities. This analysis is described in detail in Additional file 1. Our key findings with this augmented model were largely consistent with those provided in text. Specifically, we found support for the two-kernel delta-rule model, with the delta-rule model the second most supported. If anything, the new analysis was more favourable to both delta-rule models than that reported in text. In our individual-difference analysis, the delta rule and two-kernel delta rule models best fitted larger proportions of participants than reported in text, although substantial heterogeneity remained.

Some caution is warranted in interpreting our additional LLO model analysis. First, as explained in Additional file 1, two of the candidate learning models could not be meaningfully augmented—the model that samples memories (single experiences) according to delta-rule weights and the no updating model. Second, the additional LLO parameters posed some challenges to model fitting when combined with learning parameters, with potentially pathological results in some instances (see Additional file 1). Thus, although our supplementary analysis was reassuring in converging on our major conclusions, we focus primarily on our original analyses in drawing conclusions.

## Discussion

We found that learning of automation reliability was generally best described by a two-kernel delta-rule model. The delta rule accurately describes human learning in a range of domains (Lee et al., 2020). Thus, our findings connect the learning of automation reliability with learning in the broader cognitive science literature. Varying learning rates, modelled in our study by a two-kernel delta learner, can be required when there are rapid shifts in latent states (e.g., McGuire et al., 2014), as there were in the modelled Hutchinson et al. (2022a, 2022b) studies, and thus our findings in favour of variability in learning rates are also consistent with broader cognitive science literature. 

Although there is robust evidence for delta-rule like learning in many cognitive paradigms, its role has been recently debated in the context of probability perceptions, and this has implications for judgements of automation reliability. Gallistel et al. (2014) suggested that probability learning was best captured by an IIAB process rather than delta-rule learning. Although we found support for the IIAB model for subsets of participants, our findings favoured the two-kernel delta rule above the IIAB model overall. One difference between our study and Gallistel et al. is the default response. In their study, the default response (starting position of probability slider) was the judgement the participant had most recently submitted (i.e., the last judgement). This design feature has been argued to have discouraged participants from submitting smaller updates to judgements of probability, in favour of occasional step-changes (Forsgren et al., 2023). Further, a recent revaluation of Gallistel et al. indicated that perceptions of probability could be best accounted for with delta (or two-kernel delta) rule learning when this task feature was properly accounted for Forsgren et al. (2023). In Hutchinson et al. (2022a, 2022b), the default reliability judgement (starting position of the slider) was reset to 50% before each new judgement. This was implemented to encourage participants to always respond with their most recent reliability judgement, which may explain our findings in favour of (two-kernel) delta-rule learning. 

Interestingly, the two-kernel delta-rule model outperformed all memory sampling models across most of Hutchinson et al.’s (2022a, 2022b) experimental conditions. Two of the memory sampling models we tested could conceivably have explained the recency effects observed in the Hutchinson et al. data, and hence seemed at least qualitatively viable. This included the memory sampling model in which a single previous automation experience was sampled for each judgement with probabilities taking the same form as the weights in the delta rule, and the memory sampling model where either the most recent automation performance or the average automation performance was sampled. 

As recency-weighted memory sampling has outperformed the delta rule in previous studies where they were compared (e.g., Bornstein et al., 2017), support for delta rule versus memory sampling models appears to depend on specificities of task paradigms. For example, Bornstein et al. focused on a paradigm where rewards changed according to a random walk, and it has been argued this is particularly conducive to memory sampling (Ez-zizi et al., 2023). The similarities between recency-based memory sampling and delta rule (i.e., memory averaging) models may be more important than their differences. The key distinction is that memory sampling models assume small numbers of previous experiences are sampled for any given judgement. This implies variability in the effects of previous experiences on behaviour: the previous experiences that do happen to be sampled have large effects on the judgement, and experiences that are not sampled have no influence (Bornstein et al., 2017). However, both types of models imply similar average effects of past experiences on the current judgement. Indeed, the memory sampling model with sample probabilities that match delta rule weights becomes asymptotically equivalent to the delta-rule model if many memories are sampled and then averaged to form each judgement (Bornstein et al., 2017). 

The contingent sampling model provided a very poor fit to judgements of future automation reliability. A key reason for this was the model’s inability to fit the strong positive effect of the most recent observed automation accuracy on the subsequent reliability judgement (Hutchinson et al., 2022a, 2022b). The reason that the contingent sampling model could not fit the recency effect in participant reliability judgements is that automation’s *true* reliability was not positively associated with the accuracy of the most recent automation judgement. In fact, because there was a pre-set number of automation-correct contacts per trial, there was coincidentally a negative correlation between the automation’s performance on the previous contact and on the subsequent contact. In the contingent sampling model, participants remember recent events (e.g., the automation was recently correct) and predict reliability based on what followed identical sequences of events in the past (e.g., automation performance for the contact after automation was just correct). Because sequences of events in participants’ past automation experiences were not consistent with a recency effect in terms of true automation reliability, and the contingent sampling model uses these sequences to predict automation reliability, it could not simulate the recency effect observed in participants’ automation reliability judgements. 

A Bayesian model that assumed automation reliability was unchanging and attempted to learn the single “true” reliability (Wang et al., 2018) also provided a poor account of judgements of automation reliability, being generally low ranking in terms of group model comparison and providing the best model of only a small number of participants’ data. This model cannot fit substantial recency effects—in which recent automation accuracy has a larger influence on reliability judgements than automation accuracy from further in the past—as it assumes that the value of all previous experiences are equally weighted. Such recency effects, which we observed strongly in the Hutchinson et al. (2022a, 2022b) data, would be adaptive in a wide range of dynamic task environments where recent automation accuracy is more relevant to future automation accuracy than events further in the past (i.e., positive autocorrelation). For example, in the case of an adversarial attack on an automated decision aid system, after which reliability was poor, a strong recency bias would allow an operator to quickly adapt to the new automation reliability even if they had extensive experience with it performing reliably. In contrast, a “static” Bayesian model would indicate operators would adapt very slowly if they had extensive positive experience with automation, and thus would signal an inherent work system vulnerability in dynamic environments.

There was heterogeneity across participants in learning. Although the two-kernel delta-rule model was supported for the most participants of any model overall, we found non-trivial proportions of participants whose judgements were most consistent with the one-process delta-rule model, the two recency-based memory sampling models, the IIAB model, and the no-learning model. Relatively few participants submitted a series of judgements of automation reliability consistent with the Bayesian learning model or the contingent sampling model. Support for the no-learning model is perhaps the least interesting aspect of our individual differences, as it could be explained by some participants not attempting to learn about the automation reliability. In contrast, the observed heterogeneity across different learning processes is informative, because it implies that participants can adopt a range of strategies to learn about automation reliability. An important future direction will be to model the underlying causes of the heterogeneity in learning processes. For example, variation in learning processes might be framed in terms of dual-process frameworks of learning, in which people can switch between flexibly adapting, more explicit and resource-intensive learning processes, and more slowly adapting, implicit and less resource-intensive learning processes, as a function of factors such as available cognitive resources, task demands/complexity, and learning goals (e.g., Daw et al., 2011; Dienes et al., 1999; Reber, 1993; Sun et al., 2005).

The computational modelling approach that we have tested in this study provides a framework which could be applied to understand how specific task characteristics elucidate greater heterogeneity in learning processes. For example, our task included relatively few automation experiences (at maximum, 160 per participant), whereas studies that have supported the IIAB model tend to include thousands of experiences per participant (e.g., Gallistel et al., 2014). After extensive experience using automation for a certain task, expert operators likely become more confident about their predictions of automation reliability (Carter et al., 2024). In this case, they would have less reason to access finite cognitive capacity (Navon & Gopher, 1979), or to pay the costs of information access (Gray & Fu, 2004), in order to update judgements of automation reliability unless there was a notable event or evidence of a serious problem. This might involve switching to a learning process that follows the (stepwise) IIAB model more closely. Our model framework could also help to understand the underlying cognitive mechanisms by which a range of known factors affect the learning of automation reliability (Endsley, 2017; National Academies of Sciences & Medicine, 2022). For example, learning models could be applied to understand the effects of automation transparency (Bhaskara et al., 2020; Tatasciore & Loft, 2024; van de Merwe et al., 2022), or of the difficulty of the trials that automation is observed to succeed and fail on (Madhavan et al., 2006), both of which have also been shown to influence trust in automation (Hoff & Bashir, 2015).

One important consideration regarding implications is that we focused on a single-task environment. In this study, participants’ only task was vessel classification, but in safety-critical field settings operators often need to divide attention across multiple concurrent tasks (Loft et al., 2023; Remington & Loft, 2015; Wickens et al., 2022), some tasks aided by automation and some not, which can result in higher workload. This is a key feature and underlying cause of automation use error in many workplace environments (e.g., Bailey & Scerbo, 2007; Karpinsky et al., 2018; Tatasciore et al., 2023). Differences in perceived cognitive capacity as a function of task demands could affect learning processes. For example, operators may be less likely to track automation performance and instead rely on previous judgements of reliability during periods of higher workload, essentially pausing learning by adaptively trading-off information access costs against information utility (Gray & Fu, 2004), a known strategy to manage time pressure (Boag et al., 2019; Hendy et al., 1997). Similarly, it is conceivable that human operators could satisfice (Simon, 1956; Todd & Gigerenzer, 2007) with respect to learning of automation reliability, either sampling automation reliability less and/or extracting less quality evidence from the task environment (Boag et al., 2019) in situations where they perceive the automation’s reliability to be of relatively low importance to operational success. Farrell and Lewandowsky (2000) modelled automation use in multiple tasks using a connectionist learning model, and argued that effects relating to automation complacency could be explained by operators learning not to respond in the presence of automation, and learning having to be shared across multiple tasks in more complex settings. Future work extending our model framework could specify and test mechanisms by which workload and motivational factors modulate learning processes.

As reviewed earlier, in Hutchinson et al. (2022a, 2022b) the default reliability judgement was reset to 50% before each new judgement. One potential drawback to this response method is the possibility of anchoring, in which decision makers are biased towards an initially presented value (Furnham & Boo, 2011; Tversky & Kahneman, 1974). Anchoring might dilute learning effects on reliability judgements. Future research might systematically evaluate the effects of response method, and systematic biases in response production. Encouragingly, our conclusions were confirmed by supplementary analysis allowing for possible learning-independent bias in reliability judgements (i.e., with an LLO transformation).

One important application of understanding evolving judgements of automation reliability is the implications for HAT decision making. In this study, we focused on reliability judgements, rather than resulting automation reliance/HAT performance. Examining HAT performance is more difficult, because it requires a computational model not only of the learning process, but also of the processes governing human decision making and its interaction with automated advice. Hutchinson et al. (2022a, 2022b) examined automated-advice acceptance rates (reliance) with mixed-effects models, and their findings pointed to some interesting similarities and differences between patterns in automation reliance and judgements of reliability. However, they did not specify a process model of how learning automation reliability affects automation reliance.

Future research should strive to unify models of learning of automation reliability with models of automation acceptance, and understand the relationship between the two. One precedent is in Wang et al. (2022a, 2022b), who modelled automation acceptance decisions with two components, utility evaluation and action selection. They considered a range of alternative models of how humans, having been given reliability information, estimate the utility associated with accepting or rejecting automated advice. They also considered alternative models of action selection, the subsequent, utility-informed, stochastic process of accepting or rejecting automation. In Wang et al.’s study, participants were provided information about automation reliability, rather than required to estimate it. A natural extension could be to incorporate an initial model of how humans estimate automation reliability from experience (e.g., with the two-kernel delta rule), rather than providing participants with reliability information (i.e., from description). The effects of learning automation reliability from experience may differ from the effects of descriptive reliability estimates, consistent with the “description-experience gap” (Wulff et al., 2018) observed for other types of probability information.

Another approach to integrating models of learning and automation acceptance could be to build learning processes into the decision model of HAT presented by Strickland and colleagues (Strickland et al., 2021, 2023). They proposed an evidence accumulation model of how humans combine their own processing of task inputs with decision-aid inputs, which accurately describes the accuracy of human decisions and response times when they use automation. The models in this paper could inform a “front end” to the evidence accumulation process (e.g., with learning higher reliability increasing evidence accumulation in favour of agreeing with automation).

In this study, we focused on situations where automation accuracy did not depend on task features (i.e., it was random with respect to the features of the vessels that automation correctly or incorrectly classified). This emulates situations where the human has little insight into the inputs that the automation uses, or the way in which the inputs are used. However, in many circumstances, humans may rely on a mental model of how reliably automation performs with respect to task features or other context. For example, a doctor may be aware that an algorithmic recommendation performs poorly for patients with particular symptoms. Recent work by Bansal et al. (2019) examined human mental models of automation in terms of “error boundaries”, that is combinations of task features for which automation is predicted to err. Error boundaries were defined in terms of two dimensions: parsimony (i.e., how simple vs complex are the rules governing error conditions) and stochasticity (e.g., under failure conditions, does automation err every time or only sometimes). Both factors affected HAT performance, as did overall task dimensionality (the number of features the underlying task depended on). It may be fruitful for future work to unify our approaches. Although Bansal et al. examined the progression of human learning of error boundaries (e.g., relative to optimality), they were not focused on testing cognitive models of the underlying learning processes. The probability learning processes we consider here may have implications for learning of stochastic boundaries, where automation errs probabilistically.

## Conclusions

We compared a range of alternative models describing how humans learn about the reliability of automation. Across three experiments and 240 participants, we found that time series of automation reliability judgements were most consistent with a two-kernel delta rule, in which participants learned according to prediction error, with a rate that could potentially change in response to rapid changes in state. This finding is consistent with the broader success of delta rules in describing human learning. However, we also found evidence of heterogeneity in learning processes across participants, the causes of which await further investigation.

### Supplementary Information

**Additional file 1. **Supplementary materials, figures and tables.

## Data Availability

The data and computational modelling code associated with the manuscript are available at: https://osf.io/z4c5h/.

## Abbreviation

HAT: Human-automation teaming

## Footnotes

1 This value was assumed to be the latent mean of a truncated normal distribution, as with the other learning models. We also considered an alternative Bayesian response model which sampled the posterior distribution of belief to determine reliability judgements. However, this response model performed poorly, as discussed in the supplementary materials. It also required a minor transformation of the data, which complicates comparability across approaches, so it is not discussed further in text.

2 We thank one of our anonymous reviewers for this suggestion.

## Transparency and openness

The data and code specifying the computational models are available at: https://osf.io/z4c5h/. None of the experiments were preregistered.
