# Supplementary material for: How do humans learn about the reliability of automation?
Source: Cogn Res Princ Implic. 2024 Feb 16;9:8. doi: 10.1186/s41235-024-00533-1 (PMC10869332; doi:10.1186/s41235-024-00533-1)
Supplement: Supplementary file 1 — Additional file 1. Supplementary materials, figures and tables. [file 41235_2024_533_MOESM1_ESM.docx]

*How do humans learn about the reliability of automation?*

Supplementary Materials

**Contents**

- Page 2-6: Model Visualizations
- Page 7-8: Model-Fitting Procedure, Parameter Bounds and Start-Point Settings
- Pages 9-11: Alternative Response Model for Bayesian Learning
- Pages 12-14: Model Fits to Intra-Trial Variability in Judgements
- Pages 15-17: Model fits to the discrepancy between reliability judgements and empirical intra-trial reliability
- Pages 18-23: Fits of Competing Alternative Models to Reliability Judgements
- Pages 24-26: Parameters of Alternative Models
- Pages 27-30: Exploring Model Comparison Result for the Start-Low Condition in Experiment Two
- Pages 30-34: Exploration of Why the Two-Kernel Delta-Rule Outperformed the Delta Rule Model
- Pages 35-36: Statistical Comparisons of Model Parameters Across Experimental Conditions
- Pages 37-47: Exploring Bias in Probability Estimation (Linear in Log Odds Transformation)
- Page 48: References

# Model Visualizations

The plots below depict simulated predictions from each of the learning models, which may facilitate reader intuition. All models are visualized with respect to learning automation reliability in a single example, where true reliability is 50%. The initial expected reliability (prior to experience) was set to 75% for each model. Other parameter values were chosen to elucidate key features of the models. We depict the learning process as it could proceed for a single participant with effectively no observation noise (σ = 0.001), to focus purely on visualizing the learning processes.

**Figure S1**

*Simulated Example of Learning Automation Reliability According to the Bayesian Model.*


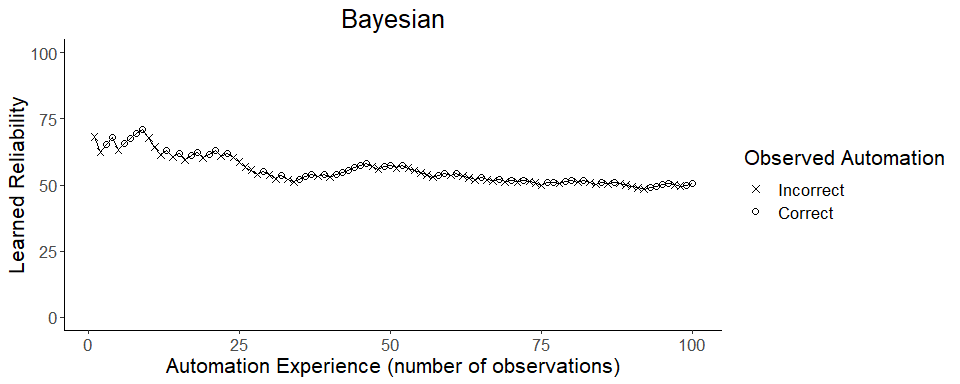


*Note.* Depicts learning from an example with true reliability of 50%. The Bayesian model slowly adapts to true reliability. Because it assumes there is a static “true” latent reliability, the more data it has already observed the less an additional data point will adjust its predictions. Thus, learning slows over the course of experience.

**Figure S2**

*Simulated Example of Learning Automation Reliability According to the Delta Rule Model*.


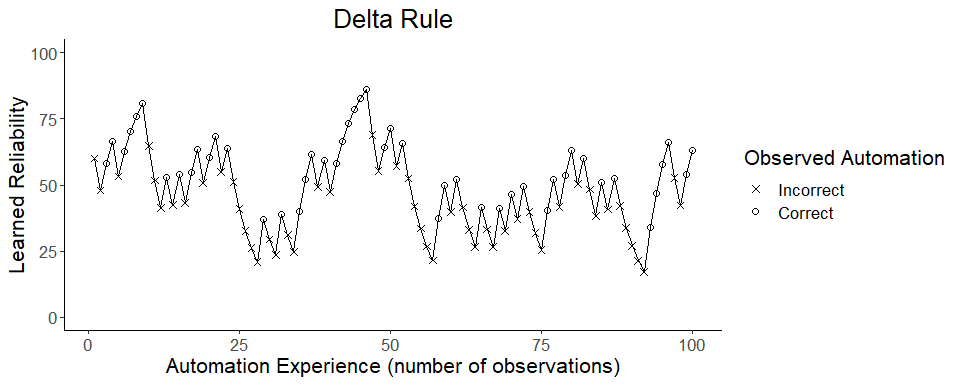


*Note.* Depicts learning from an example with true reliability of 50%. This model learns from each automation experience proportional to prediction error but cannot adjust its learning rate. Although it adapts towards the true reliability of 50%, reliability estimates remain volatile.

**Figure S3**

*Simulated Example of Learning Automation Reliability According to the Two-Kernel Delta Rule Model.*


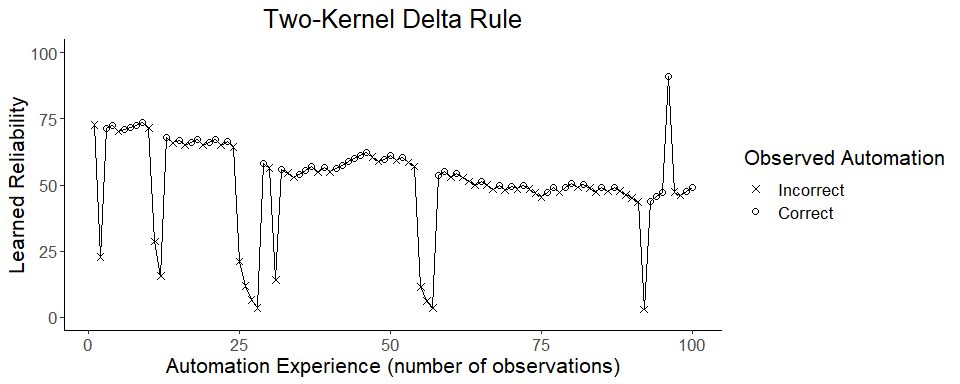


*Note.* Depicts learning from an example with true reliability of 50%. Two kernels adapt, one slowly and one quickly, with the fast kernel determining output under environmental volatility. This model produces slow-running average adaptations, but with potentially strong recency effects at times.

**Figure S4**

*Simulated Example of Learning in the Model That Samples Memory According to Delta Rule Weights*.


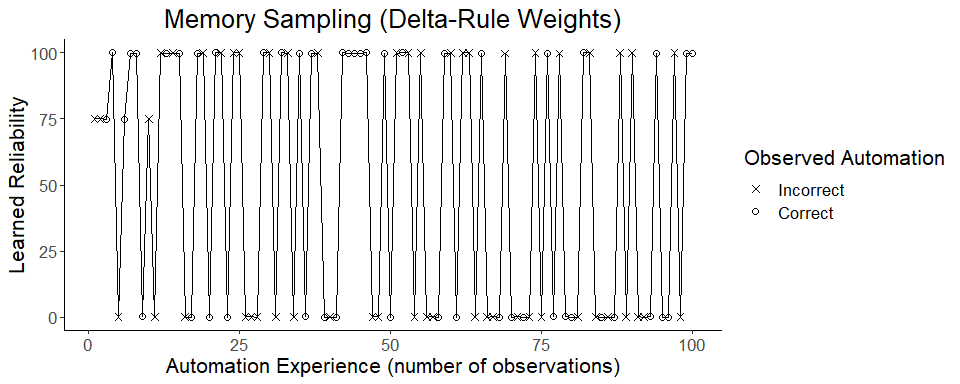


*Note.* Depicts learning from an example with true reliability of 50%. The probability of sampling previous experiences is recency-weighted, with weights matching those used for recency-weighted averaging in the delta rule.

**Figure S5**

*Simulated Example of Learning in The Model That Samples Either the Last Automation Experience, or the Total Average*

*
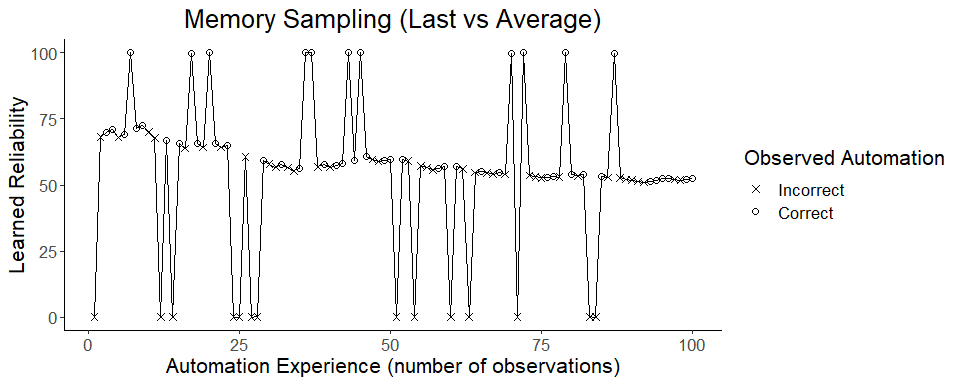
*

*Note.* Depicts learning from an example with true reliability of 50%. The model either samples the last automation experience (which is either correct or incorrect), or the average of all experience (and prior belief).

**Figure S6**

*Simulated Example of Learning in the Contingent Sampling Model*.


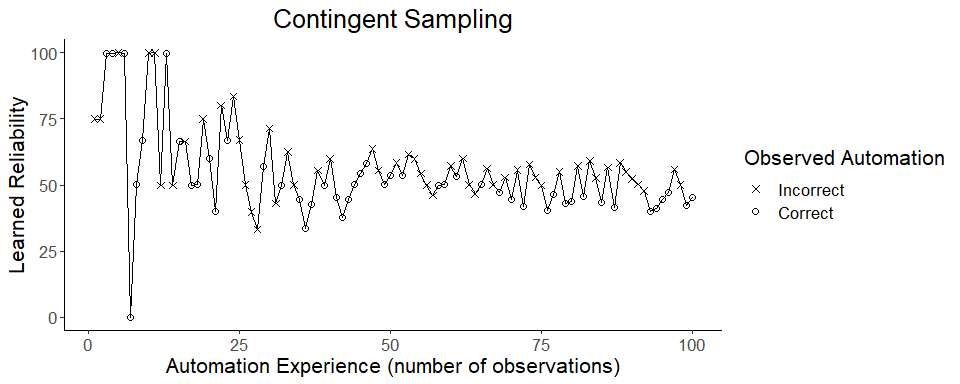


*Note.* Depicts learning from an example with true reliability of 50%. Simulated with *m* = 2. The initial judgements (for experience < *m*) are set to *r0_sampling_contingent_* (here, 75%). Once experience ≥ *m,* memories with immediate histories matching the previous *m* experiences are used to determine reliability judgements. Where experience ≥ *m* but there are no matching samples of experience, a random sample is drawn for prediction. Where possible, matching samples of experience are drawn (this is the primary learning mechanism of the model). As instances of memory accrue, this learning mechanism begins to adapt accurately (i.e., converges towards 50% in this example).

**Figure S7**

*Simulated Example of Learning from the IIAB Model*

#
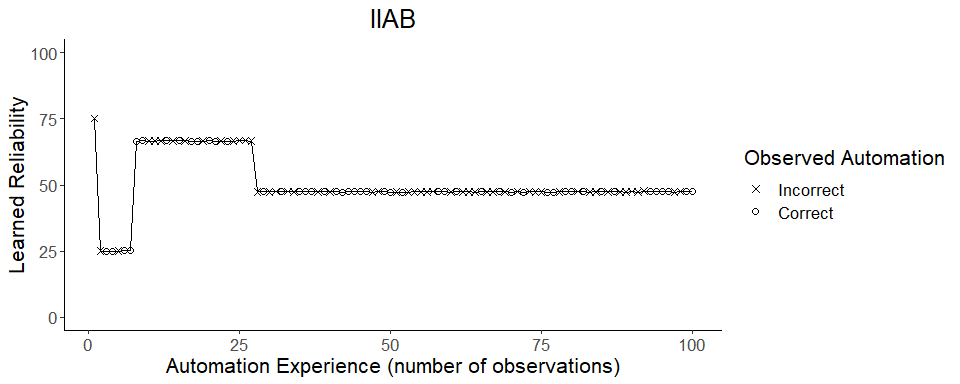


*Note.* Depicts learning from an example with true reliability of 50%. A key feature of the model is step learning. In this example, it adjusts with experience towards an estimate of 50% reliability, but does not adapt further once getting close (it would require more evidence to do so).

# Model-Fitting Procedure, Parameter Bounds and Start-Point Settings

## Model parameters were estimated by applying the bounded Nelder-Mead optimization algorithm, as implemented in the “dfoptim” R package (Varadhan et al., 2020), to minimize the negative log-likelihood function for each model. This algorithm, accessed with the ‘nmkb’ function, was run with default settings except that the number of evaluations was set to 5000 (rather than 1500) and maximum number of restarts set to 10 (rather than 3).

## Parameter bounds are available in Table S1. To mitigate potential issues with local minima, we tried fitting each model with a range of different start-points for its parameters, and ultimately selected whichever fit was the best for each model (i.e., lowest negative log likelihood). For each parameter we defined a parameter range (in Table S1) and picked 10 equally spaced points within that range. We then created a grid of all combinations of these possible model parameter values. We sampled 200 sets of start points randomly from that grid for all models except for the no updating and the contingent sampling models. For those models, there were only two continuous parameters ($\boldsymbol{r}_{\mathbf{0}}$ and σ), and hence only 100 possible start-point value combinations, all of which were tested. In addition to the automatically generated grids of start points under consideration, we tried fits with one hand-picked set of start points for each model (Table S1).

The contingent sampling model also included a discrete parameter *m* that could not be estimated with continuous methods. Instead, we exhaustively ran fits for every discrete value of this parameter within a plausible range (1-10), optimizing the other two parameters (*r0_sampling_contingent_* and σ) in the usual way, and chose the fits with the highest likelihood as the final parameter values.

In all cases, the selected model fits converged, as defined by the default tolerance of 10^-6^ in the dfoptim package.

**Table S1**

*The parameter bounds and ranges of start points that were sampled for each model. We initially hand-picked start points (rightmost column). To address the possibility of local optima also tried sampling start points from a grid (constrained by the ranges in the second rightmost column).*

| Model | Parameter | Bounds | Start point range | Hand-picked start point |
| --- | --- | --- | --- | --- |
| All | σ | 0.01-5 | 0.02-1 | 0.1 |
| Bayesian Learning | *p* | 0-500 | 0.01-20 | 1 |
|  | *q* | 0-500 | 0.01-20 | 1 |
| Delta updating | $r_{0}$ | 0-1 | 0.01-0.99 | 0.5 |
|  | α | 0-1 | 0.01-0.99 | 0.2 |
| Two-kernel Delta updating | $r_{0}$ | 0-1 | 0.01-0.99 | 0.5 |
|  | α_slow_ | 0-1 | 0.01-0.99 | 0.2 |
|  | α_fast_ | 0-1 | 0.01-0.99 | 0.2 |
|  | *T* | 0-1 | 0.01-0.99 | 0.1 |
| Memory Sampling (delta-rule weights) | *r0_sampling_recency_* | 0-1 | 0.01-0.99 | 0.5 |
|  | α*_sampling_* | 0-1 | 0.01-0.99 | 0.2 |
| Memory Sampling (recent/average) | *r0_sampling_last_average_* | 0-1 | 0.01-0.99 | 0.5 |
|  | *prob_last_* | 0-1 | 0.01-0.99 | 0.5 |
|  | *weight_r0_* | 0-500 | 0.01-100 | 1 |
| Contingent Sampling | *r0_sampling_contingent_* | 0-1 | 0.01-0.99 | 0.5 |
|  | *m (discrete parameter)* | 1-10 | 1-10 |  |
| IIAB | *p* | .001-100 | 0.01-20 | 1 |
|  | *q* | .001-100 | 0.01-20 | 1 |
|  | $p_{change point}$ | .001-100 | 0.01-20 | 0.5 |
|  | $q_{change point}$ | .001-100 | 0.01-20 | 0.5 |
|  | *T_1_* | 0-100 | 0.1-10 | 0.82 |
|  | *T_2_* | 0-100 | 1-20 | 2 |
| No Updating | $r_{0}$ | 0-1 | 0.01-0.99 | 0.5 |

# Alternative Response Model for Bayesian Learning

The alternative Bayesian model specified the same learning process as that described in the main text, with beliefs about automation reliability following a beta distribution. However, rather than assuming that this learning process is summarized by the posterior mean, and observation noise obtained from a truncated normal distribution, the alternative model assumes that noise results from participants sampling from their posterior distribution of beliefs. For this model, beta likelihoods are not defined for values of exactly 0 or 1, and so we had to slightly transform the reliability judgements before fitting this model: *New Rating = (Future Reliability/100 - .5) x 0.99 + 0.5)*. For the purposes of comparison, we also refitted the Bayesian model discussed in the main text (where the learning process defined the latent mean to a truncated normal distribution) to this transformed data. Our analysis indicated that the response method applied in main text (posterior mean + independent noise from truncated normal distribution) was substantially more supported by BIC than the alternative response model (posterior sample) in every experiment (Table S2). Investigating the data, one apparent reason for the drastic disadvantage of the alternative posterior-sample response model was the prediction of drastically decreasing response variability over the course of each experiment (e.g., see Figure S8) due to decreased uncertainty (narrower posterior), which was not consistent with actual participant reliability judgements.

**Table S2**

*Group (summed) BIC values for the two alternative Bayesian learning models which assumed a static true automation reliability. For every condition, the model which used the posterior mean + noise from a truncated normal distribution was favoured substantially over the model that assumed response noise owed to participants sampling their posterior distribution of belief. We report BIC in terms of the difference from the most supported model for each experiment condition (hence, the most supported model for each experimental condition has a value of 0).*

|  |  |  | Experiment | | |  |  |  |
| --- | --- | --- | --- | --- | --- | --- | --- | --- |
|  | One | | Two | | | Three | | |
| Bayesian Learning Response Model | High | Low | Start-High | Start-Low | Constant | Large Drop | Medium Drop | Small Drop |
| Mean + Noise | 0 | 0 | 0 | 0 | 0 | 0 | 0 | 0 |
| Posterior Sample | 27678 | 24069 | 48502 | 61084 | 40627 | 55426 | 31028 | 17031 |

**Figure S8**

*Experiment 1, Model Fit to Intra-Trial Standard Deviation (Averaged Across Participants) of Each Block*.


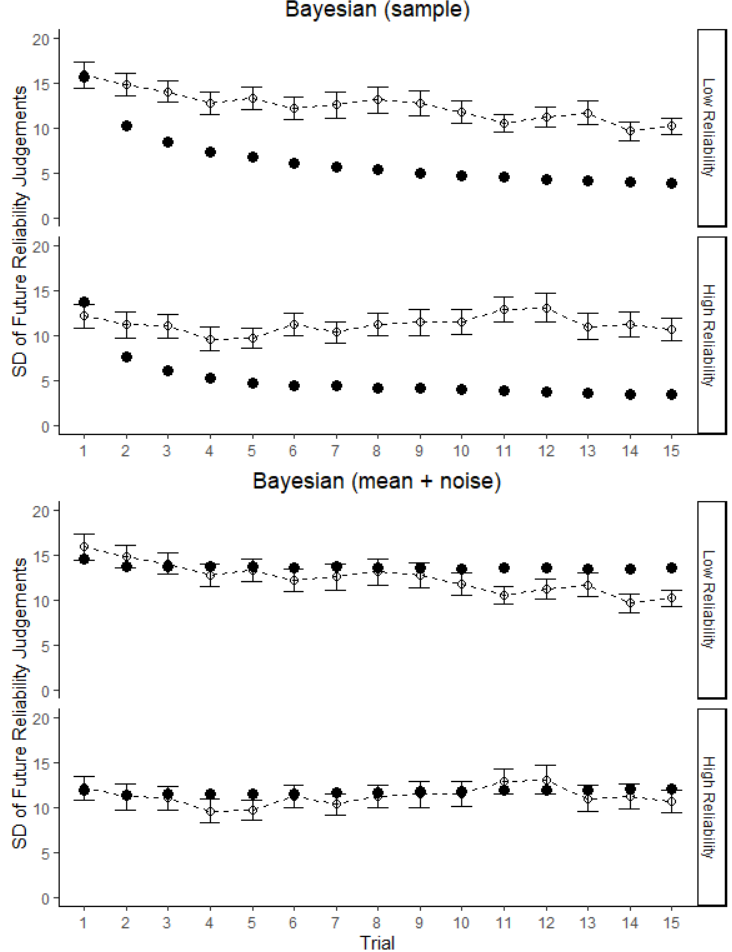


*Note.* The data corresponds to the white circles, the model mean predictions to the black dots. The error bars display the data means plus or minus the standard error. The top plots correspond to the Bayesian model reported in text, and the bottom plot to the alternative formulation which assumes judgements are a random sample of the posterior.

**Model Fits to Intra-Trial Variability in Judgements**

**Figure S9**

*Experiment 1, Fit of the Two-Kernel Delta Rule to Intra-Trial Standard Deviation (Averaged Across Participants) of Each Block*.*
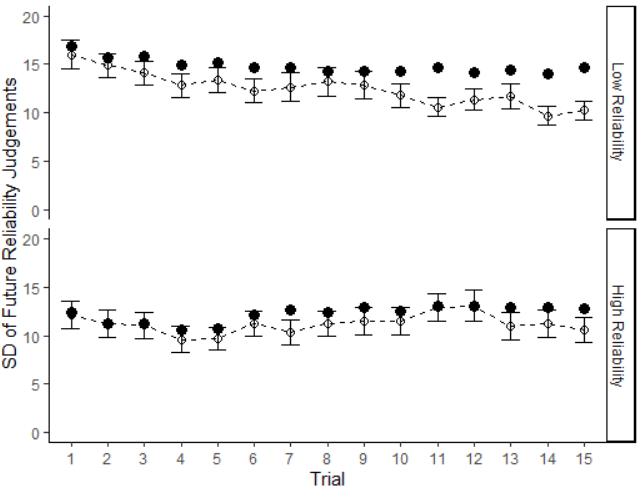
*

*Note.* The data corresponds to the white circles, the model mean predictions to the black dots. The error bars display the data means plus or minus the standard error.

**Figure S10**

*Experiment 3, Fit of the Two-Kernel Delta Rule to Intra-Trial Standard Deviation (Averaged Across Participants) Of Each Block.*


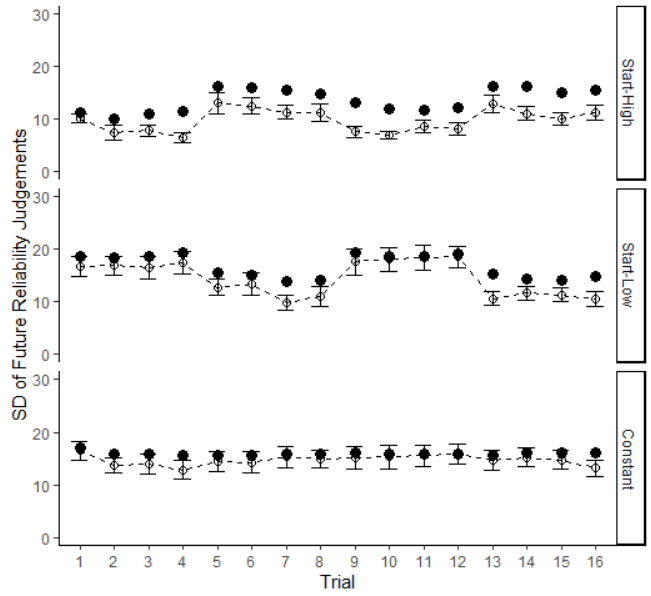


*Note.* The data corresponds to the white circles, the model mean predictions to the black dots. The error bars display the data means plus or minus the standard error.

**Figure S11**

*Experiment 3, Fit of the Two-Kernel Delta Rule to Intra-Trial Standard Deviation (Averaged Across Participants) Of Each Block.*


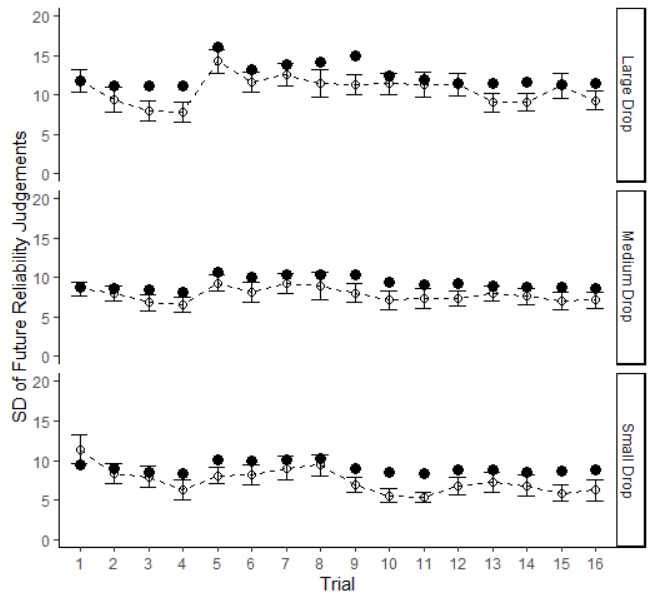


*Note.* The data corresponds to the white circles, the model mean predictions to the black dots. The error bars display the data means plus or minus the standard error.

**Model fits to the discrepancy between reliability judgements and empirical intra-trial reliability**

**Figure S12**

*Experiment 1, Fit of the Two-Kernel Delta Rule to the Discrepancy Between Automation Reliability Judgements and Empirically Observed Intra-Trial Automation Accuracy*


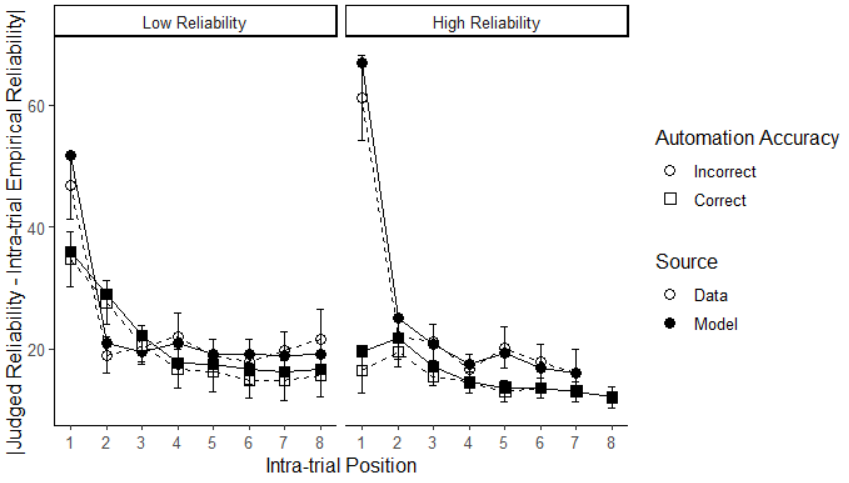


*Note.* Plots the absolute difference between participant reliability judgements and the average automation accuracy that had been observed within each trial up until the relevant intra-trial position. For example, for intra-trial position 1 the participant had only empirically observed automation once within that trial, and thus empirically observed intra-trial accuracy would be either 100% or 0%. Discrepancies were particularly large for intra-trial position 1 when automation was incorrect, because participant reliability estimates were usually much larger than 0. The data corresponds to the white circles, the model mean predictions to the black dots. The error bars display the data means plus or minus the standard error.

**Figure S13**

*Experiment 2, Fit of the Two-Kernel Delta Rule to the Discrepancy Between Automation Reliability Judgements and Empirically Observed Intra-Trial Automation Accuracy*

*
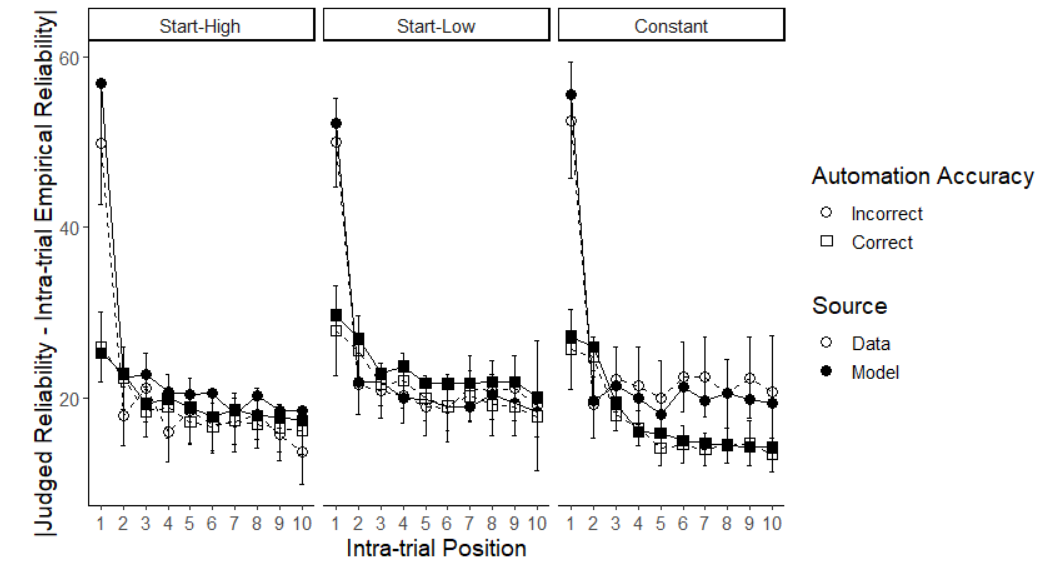
*

*Note.* Plots the absolute difference between participant reliability judgements and the average automation accuracy that had been observed within each trial up until the relevant intra-trial position. For example, for intra-trial position 1 the participant had only empirically observed automation once within that trial, and thus empirically observed intra-trial accuracy would be either 100% or 0%. Discrepancies were particularly large for intra-trial position 1 when automation was incorrect, because participant reliability estimates were usually much larger than 0. The data corresponds to the white circles, the model mean predictions to the black dots. The error bars display the data means plus or minus the standard error.

**Figure S14**

*Experiment 3, Fit of The Two-Kernel Delta Rule to the Discrepancy Between Automation Reliability Judgements and Empirically Observed Intra-Trial Automation Accuracy.*

*
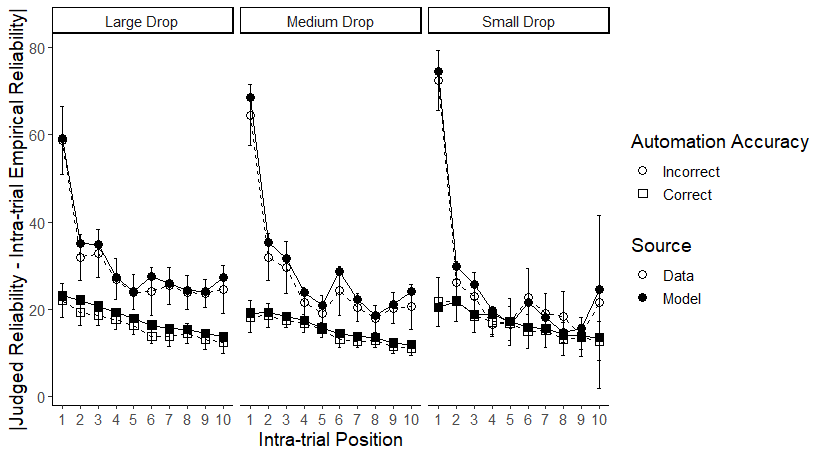
*

*Note.* Plots the absolute difference between participant reliability judgements and the average automation accuracy that had been observed within each trial up until the relevant intra-trial position. For example, for intra-trial position 1 the participant had only empirically observed automation once within that trial, and thus empirically observed intra-trial accuracy would be either 100% or 0%. Discrepancies were particularly large for intra-trial position 1 when automation was incorrect, because participant reliability estimates were usually much larger than 0. The data corresponds to the white circles, the model mean predictions to the black dots. The error bars display the data means plus or minus the standard error.

**Fits of Competing Alternative Models to Reliability Judgements**

**Figure S15**

*Averaged Predictions of the Bayesian Model for Experiments 1,2, and 3 (Hutchinson et al., 2022a; 2022b)*

##
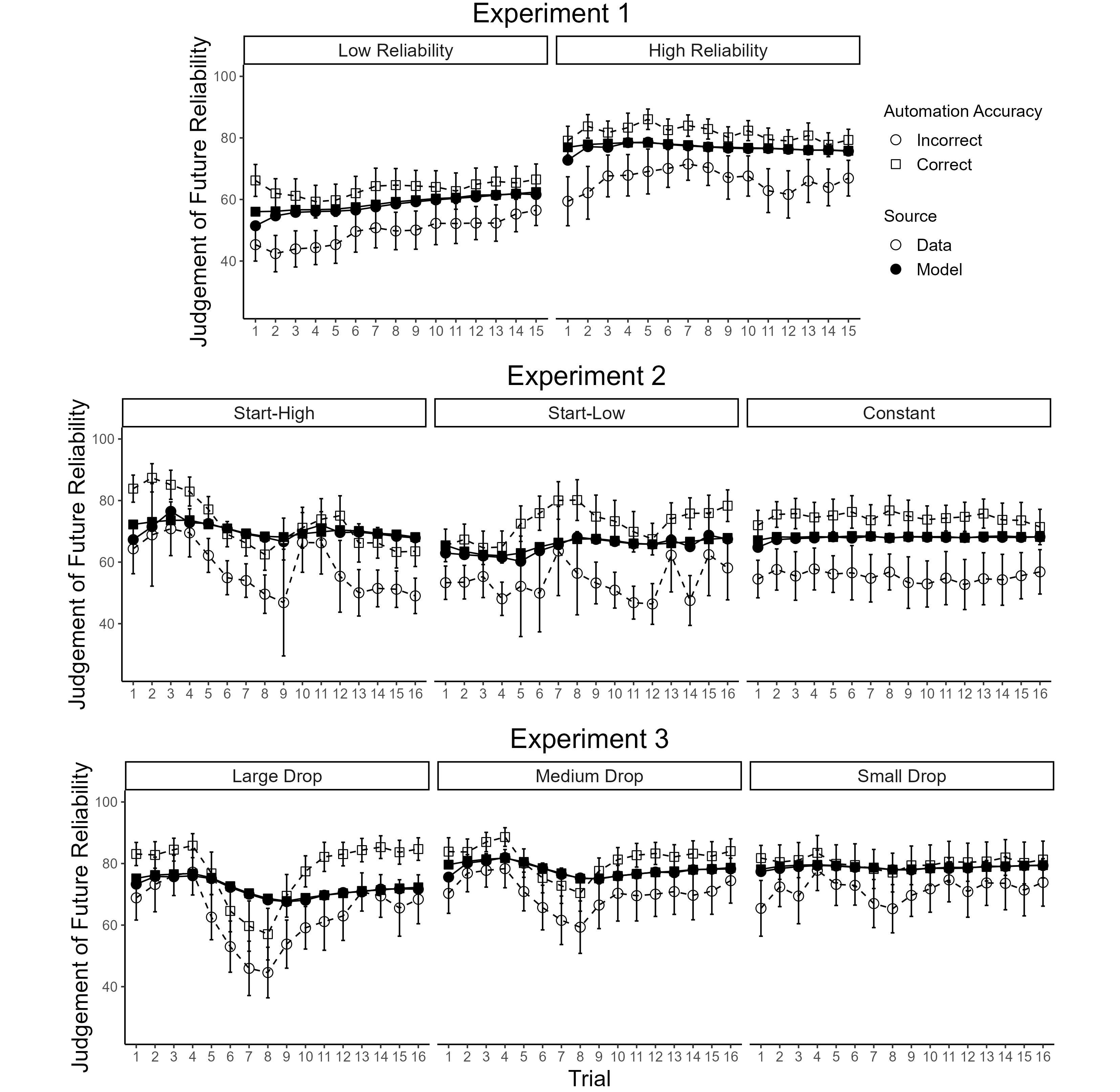


*Note*. The data corresponds to the white circles, the model mean predictions to the black dots. The error bars display the data means plus or minus the standard error.

**Figure S16**

*Averaged Predictions of the Delta-Rule Model for Experiments 1,2, and 3 (Hutchinson et al., 2022a; 2022b).*
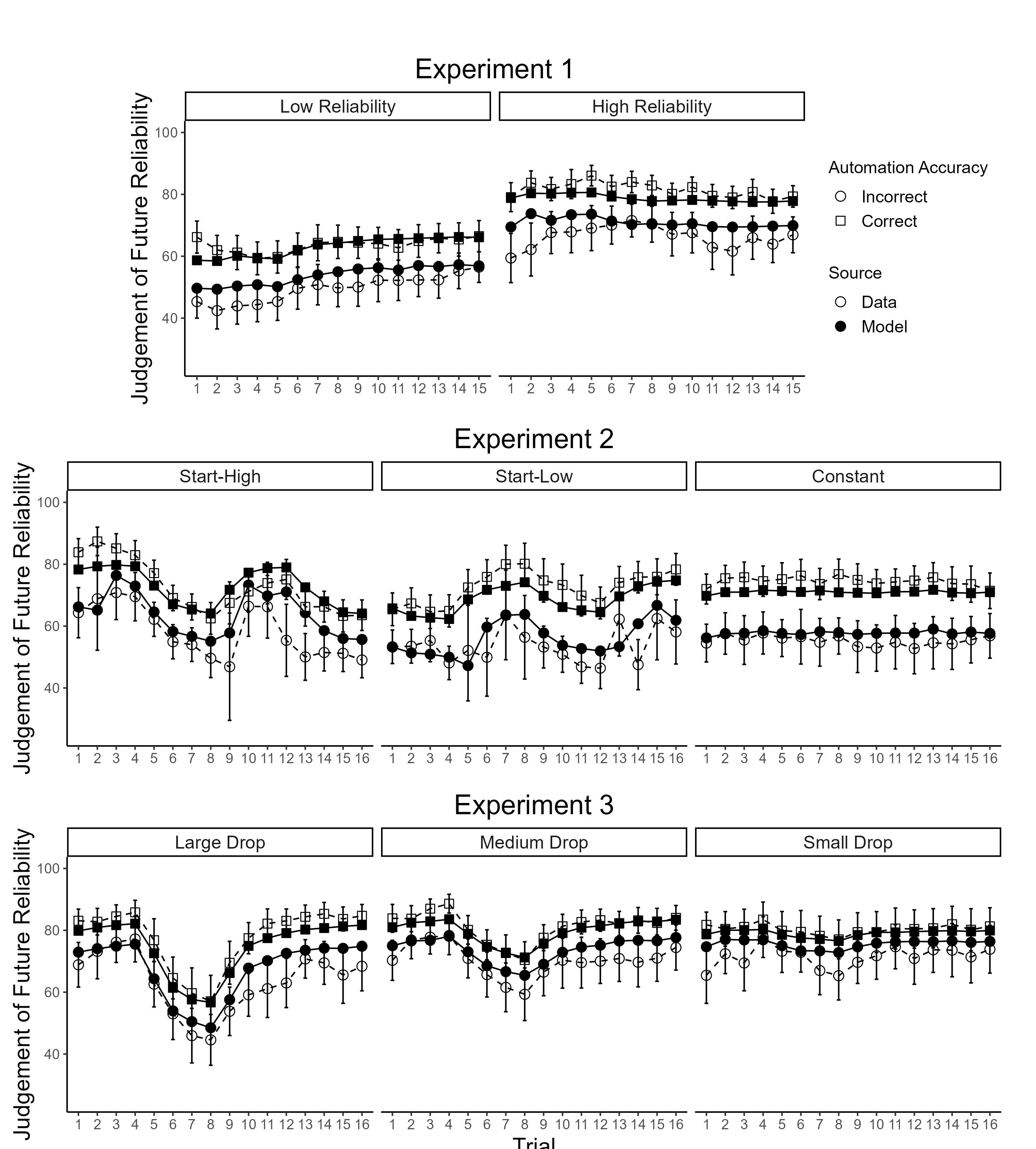
 *Note*. The data corresponds to the white circles, the model mean predictions to the black dots. The error bars display the data means plus or minus the standard error.

**Figure S17**

*Averaged Predictions of the Memory Sampling (Delta Rule Weights) Model for Experiments 1,2, and 3 (Hutchinson et al., 2022a; 2022b).*
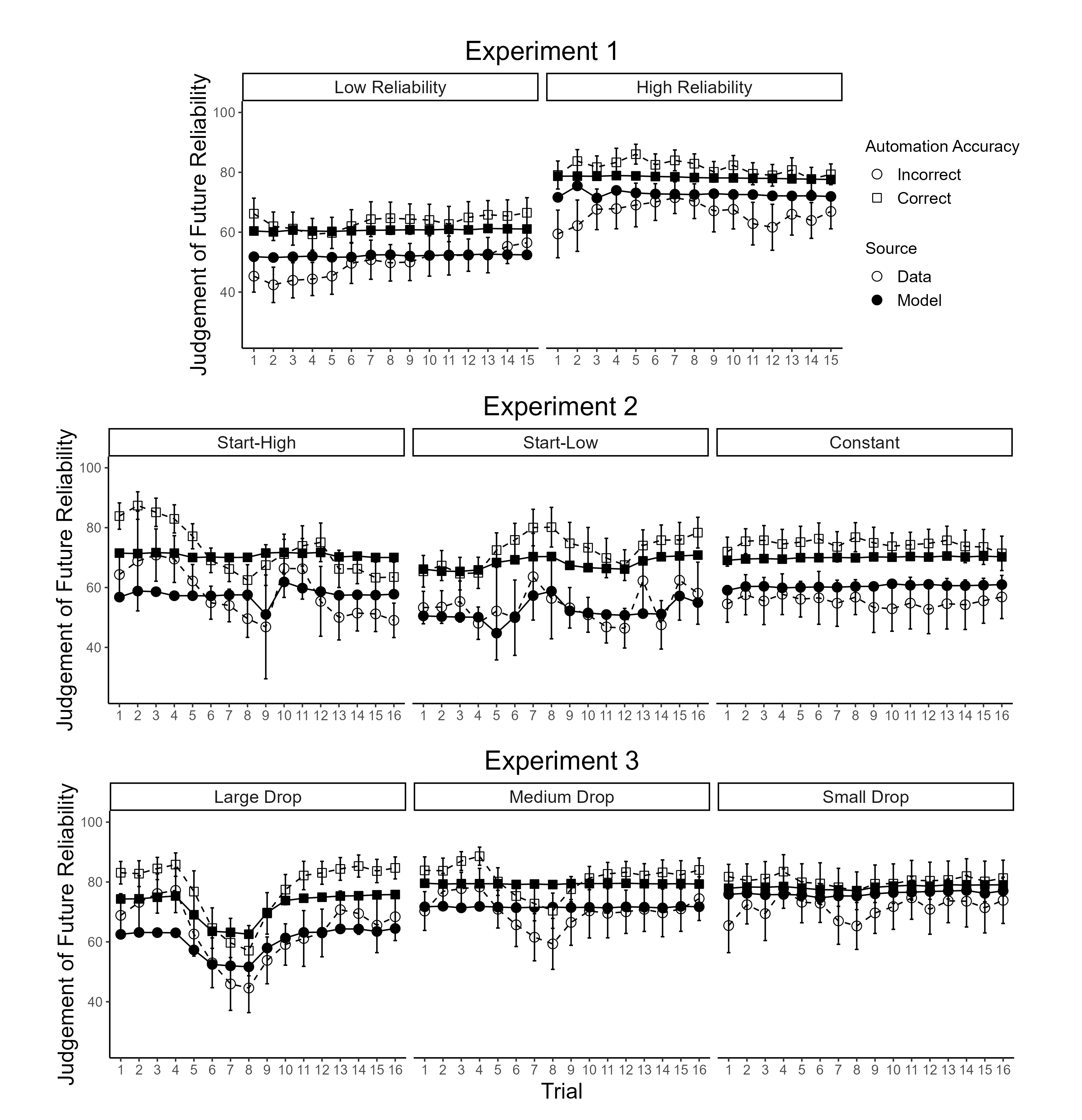


*Note*. The data corresponds to the white circles, the model mean predictions to the black dots. The error bars display the data means plus or minus the standard error.

## Figure S18

## *Averaged Predictions of the Memory Sampling (Last Experience Versus Average) Model for Experiments 1,2, and 3 (Hutchinson et al., 2022a; 2022b)*

##
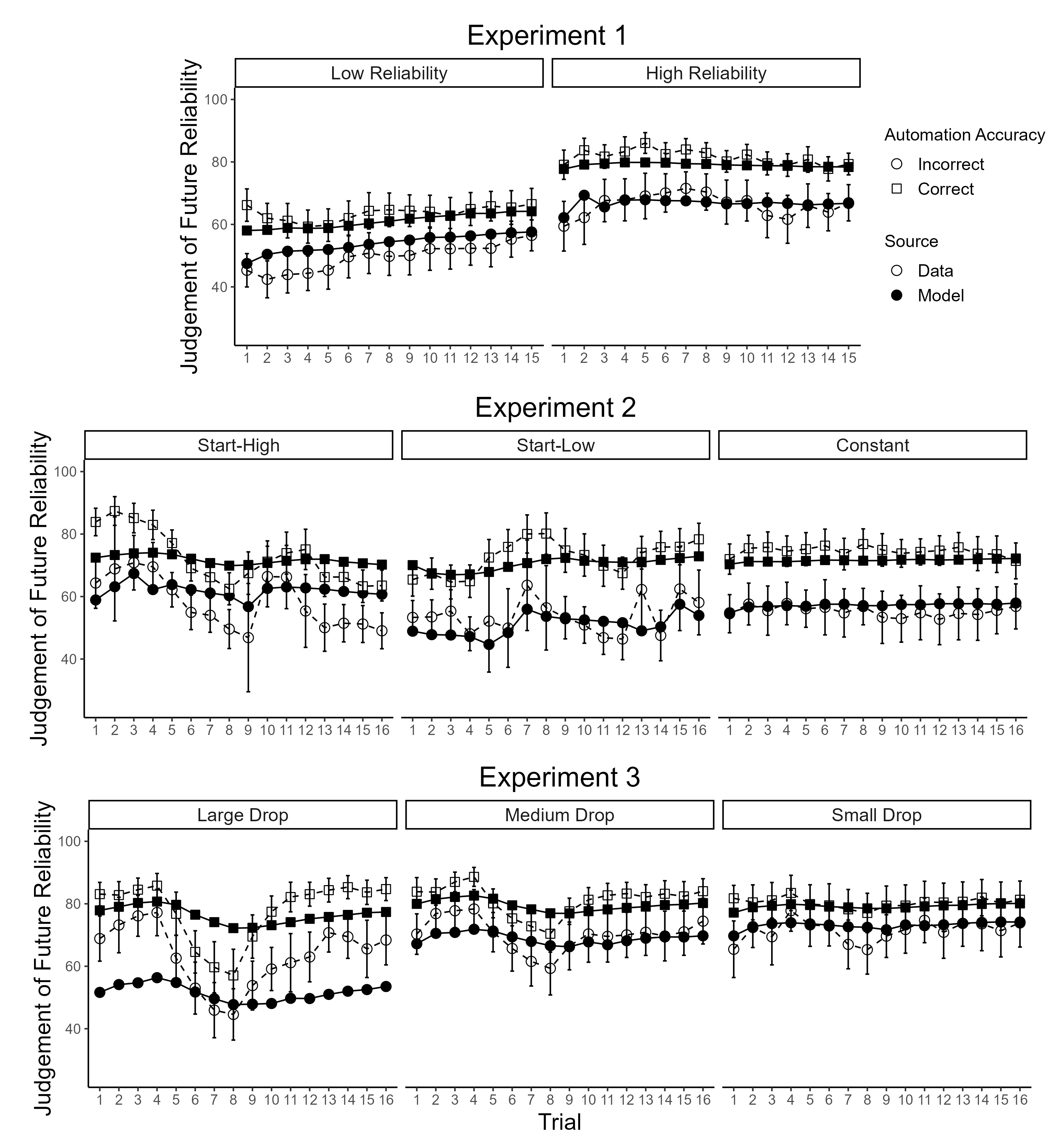


## *Note*. The data corresponds to the white circles, the model mean predictions to the black dots. The error bars display the data means plus or minus the standard error.

## Figure S19

## *Averaged Predictions of the Contingent Sampling Model for Experiments 1,2, and 3 (Hutchinson et al., 2022a; 2022b)*


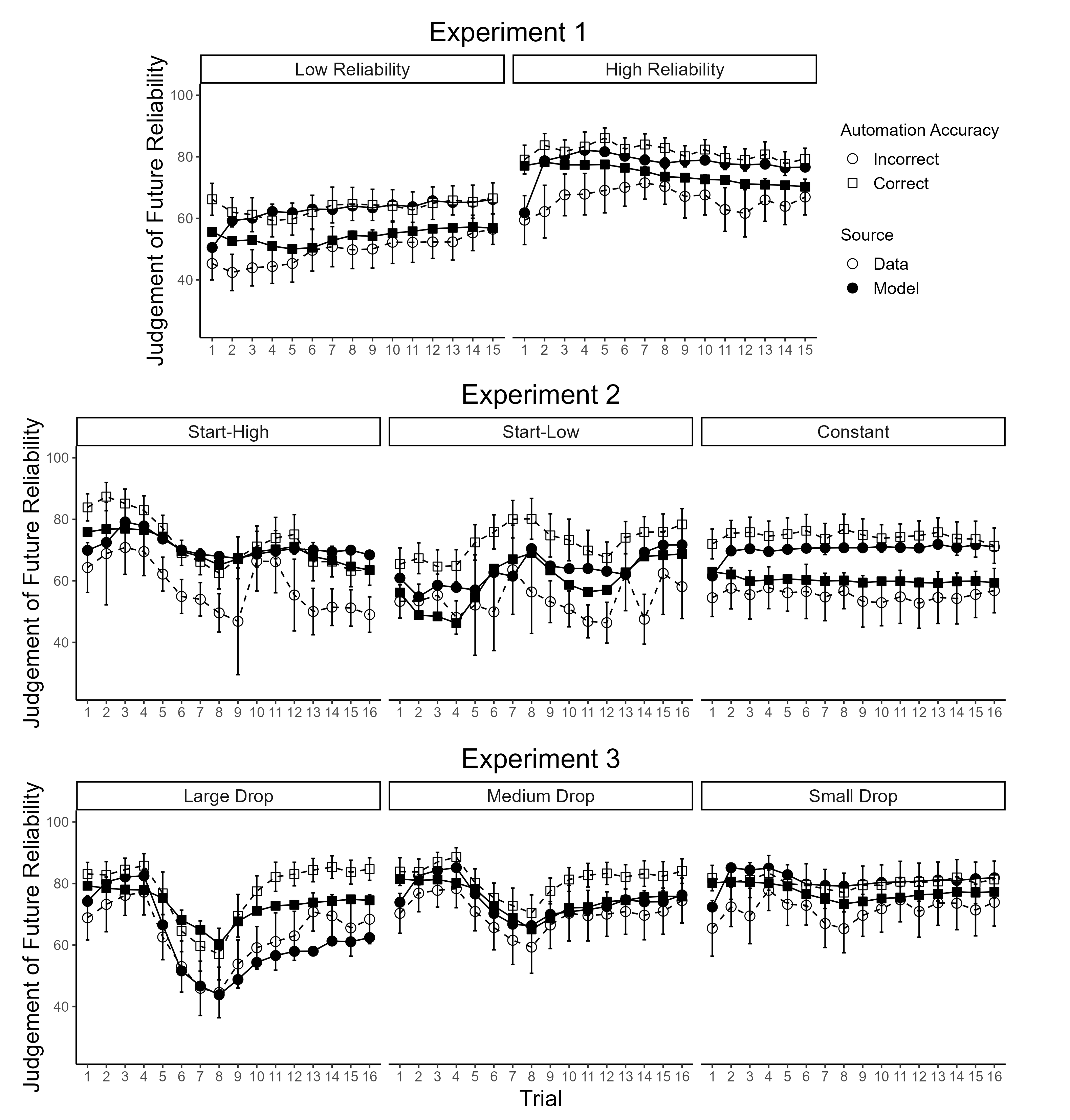


## *Note*. The data corresponds to the white circles, the model mean predictions to the black dots. The error bars display the data means plus or minus the standard error.

## Figure S20

## *Averaged Predictions of the IIAB Model for Experiments 1,2, and 3 (Hutchinson et al., 2022a; 2022b)*


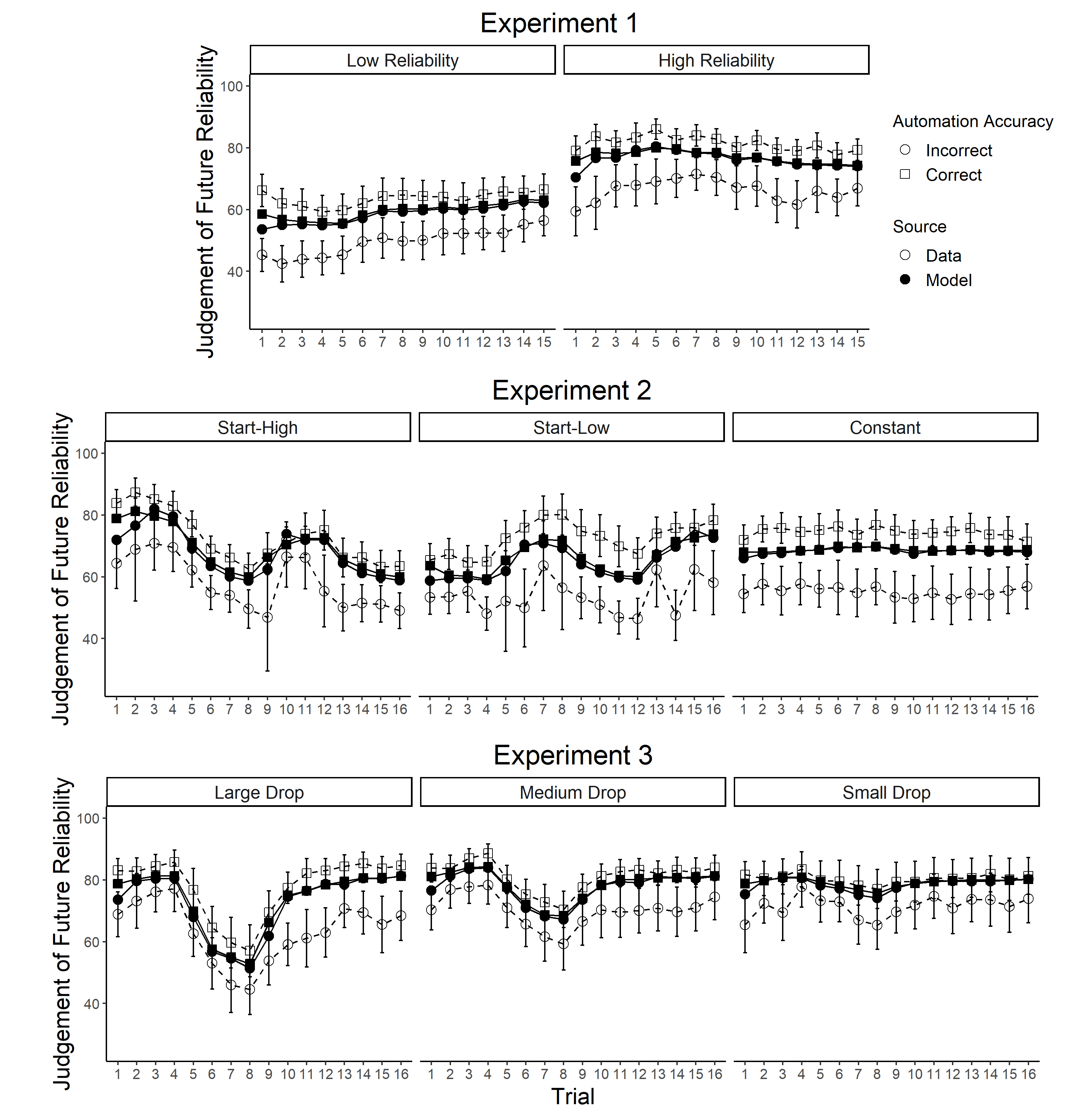


## *Note*. The data corresponds to the white circles, the model mean predictions to the black dots. The error bars display the data means plus or minus the standard error.

**Parameters of Alternative Models**

**Table S3**

*Estimated Parameter Values of the Bayesian Model, Presented as M (SE).*

| Experiment | Condition | $p$ | *q* | *σ* |
| --- | --- | --- | --- | --- |
| One | Low Reliability | 160.69 (35.44) | 115.41 (25.73) | 0.18 (0.03) |
|  | High Reliability | 265.02 (39.2) | 44.04 (14.88) | 0.17 (0.02) |
| Two | Start-High | 187.51 (44.07) | 80.84 (26.59) | 0.37 (0.17) |
|  | Start-Low | 262.37 (43.72) | 66.69 (24.94) | 0.26 (0.03) |
|  | Constant | 327.12 (43.02) | 126.69 (31.18) | 0.21 (0.03) |
| Three | Large Drop | 243.41 (46.12) | 6.37 (2.94) | 0.24 (0.03) |
|  | Medium Drop | 224.34 (46.29) | 27.97 (13.19) | 0.14 (0.02) |
|  | Small Drop | 316.74 (45.31) | 89.54 (28.57) | 0.12 (0.02) |

**Table S4**

*Estimated Parameter Values of the Delta Rule Model, Presented as M (SE)*

| Experiment | Condition | $r_{0}$ | α | *σ* |
| --- | --- | --- | --- | --- |
| One | Low Reliability | 0.58 (0.04) | 0.18 (0.04) | 0.20 (0.06) |
|  | High Reliability | 0.83 (0.04) | 0.13 (0.03) | 0.15 (0.01) |
| Two | Start-High | 0.82 (0.04) | 0.16 (0.04) | 0.32 (0.17) |
|  | Start-Low | 0.64 (0.06) | 0.23 (0.06) | 0.22 (0.03) |
|  | Constant | 0.62 (0.06) | 0.24 (0.07) | 0.17 (0.02) |
| Three | Large Drop | 0.82 (0.05) | 0.13 (0.03) | 0.16 (0.02) |
|  | Medium Drop | 0.80 (0.04) | 0.10 (0.04) | 0.11 (0.01) |
|  | Small Drop | 0.77 (0.05) | 0.06 (0.03) | 0.12 (0.02) |

**Table S5**

*Estimated parameter values of the memory sampling (according to delta rule weights) model, presented as M (SE).*

| Experiment | Condition | *r0_sampling_recency_* | α*_sampling_* | *σ* |
| --- | --- | --- | --- | --- |
| One | Low Reliability | 0.57 (0.05) | 0.23 (0.07) | 0.24 (0.06) |
|  | High Reliability | 0.87 (0.03) | 0.11 (0.04) | 0.17 (0.01) |
| Two | Start-High | 0.73 (0.06) | 0.29 (0.08) | 0.39 (0.17) |
|  | Start-Low | 0.61 (0.06) | 0.34 (0.08) | 0.23 (0.03) |
|  | Constant | 0.65 (0.04) | 0.17 (0.06) | 0.14 (0.01) |
| Three | Large Drop | 0.83 (0.03) | 0.23 (0.06) | 0.19 (0.02) |
|  | Medium Drop | 0.86 (0.03) | 0.15 (0.07) | 0.15 (0.02) |
|  | Small Drop | 0.81 (0.04) | 0.04 (0.02) | 0.11 (0.01) |

**Table S6**

*Estimated parameter values of the memory sampling model (last experience versus average of all experiences), presented as M (SE).*

| Experiment | Condition | *r0_sampling_last_average_* | *weight_r0_* | *prob_t_* | *σ* |
| --- | --- | --- | --- | --- | --- |
| One | Low Reliability | 0.56 (0.06) | 207.05 (37.67) | 0.10 (0.03) | 0.16 (0.03) |
|  | High Reliability | 0.74 (0.05) | 282.70 (38.03) | 0.17 (0.04) | 0.15 (0.01) |
| Two | Start-High | 0.67 (0.06) | 230.97 (45.59) | 0.15 (0.04) | 0.33 (0.17) |
|  | Start-Low | 0.73 (0.05) | 245.12 (41.23) | 0.26 (0.05) | 0.19 (0.03) |
|  | Constant | 0.65 (0.05) | 321.17 (41.17) | 0.19 (0.06) | 0.14 (0.01) |
| Three | Large Drop | 0.53 (0.08) | 134.50 (37.49) | 0.33 (0.05) | 0.14 (0.01) |
|  | Medium Drop | 0.77 (0.06) | 232.31 (46.62) | 0.15 (0.05) | 0.12 (0.01) |
|  | Small Drop | 0.67 (0.07) | 319.39 (44.88) | 0.07 (0.03) | 0.10 (0.01) |

**Table S7**

*Estimated parameter values of the contingent sampling model, presented as M (SE).*

| Experiment | Condition | *r0_sampling_contingent_* | *m* | *σ* |
| --- | --- | --- | --- | --- |
| One | Low Reliability | 0.65 (0.06) | 4.19 (0.68) | 0.64 (0.18) |
|  | High Reliability | 0.75 (0.04) | 2.33 (0.36) | 0.23 (0.01) |
| Two | Start-High | 0.85 (0.03) | 2.52 (0.57) | 0.42 (0.16) |
|  | Start-Low | 0.71 (0.04) | 6.62 (0.73) | 0.33 (0.03) |
|  | Constant | 0.74 (0.06) | 4.11 (0.71) | 0.55 (0.17) |
| Three | Large Drop | 0.81 (0.04) | 3.25 (0.58) | 0.22 (0.01) |
|  | Medium Drop | 0.85 (0.03) | 3.19 (0.61) | 0.18 (0.01) |
|  | Small Drop | 0.78 (0.03) | 2.08 (0.49) | 0.37 (0.20) |

**Table S8**

*Estimated parameter values of the IIAB model, presented as M (SE).*

| Experiment | Condition | $p$ | $q$ | $p_{change}$ | $q_{change}$ | *T_1_* | *T_2_* | *σ* |
| --- | --- | --- | --- | --- | --- | --- | --- | --- |
| One | Low Reliability | 32.03 (5.93) | 31.59 (5.84) | 48.32 (6.10) | 32.88 (5.86) | 13.85 (4.78) | 30.84 (6.20) | 0.16 (0.02) |
|  | High Reliability | 54.08 (7.07) | 19.12 (5.20) | 40.30 (5.72) | 35.68 (6.85) | 38.51 (7.37) | 28.46 (5.88) | 0.16 (0.01) |
| Two | Start-High | 38.18 (7.78) | 24.30 (6.98) | 52.12 (6.83) | 33.49 (6.47) | 16.56 (6.69) | 28.19 (7.02) | 0.15 (0.02) |
|  | Start-Low | 66.51 (7.97) | 24.90 (6.53) | 53.77 (6.88) | 47.08 (7.37) | 29.86 (8.30) | 28.67 (6.73) | 0.21 (0.02) |
|  | Constant | 57.75 (7.86) | 23.40(6.23) | 25.03 (6.22) | 28.49 (5.84) | 36.10 (8.22) | 22.29 (6.32) | 0.20 (0.03) |
| Three | Large Drop | 44.86 (7.24) | 12.16 (4.05) | 54.33 (7.36) | 25.91 (6.63) | 21.32 (7.68) | 32.62 (7.34) | 0.15 (0.01) |
|  | Medium Drop | 45.27 (7.78) | 13.24 (4.91) | 42.05 (7.59) | 29.48 (7.10) | 22.37 (7.46) | 30.48 (6.87) | 0.11 (0.01) |
|  | Small Drop | 54.96 (8.17) | 16.05 (5.21) | 44.07 (7.84) | 27.34 (6.97) | 37.85 (9.69) | 26.40 (6.56) | 0.11 (0.01) |

**Table S9**

*Estimated parameter values of the no updating model, presented as M (SE).*

| Experiment | Condition | $r_{0}$ | *σ* |
| --- | --- | --- | --- |
| One | Low Reliability | 0.62 (0.03) | 0.21 (0.04) |
|  | High Reliability | 0.90 (0.02) | 0.20 (0.02) |
| Two | Start-High | 0.76 (0.04) | 0.38 (0.17) |
|  | Start-Low | 0.77 (0.03) | 0.27 (0.03) |
|  | Constant | 0.78 (0.03) | 0.22 (0.03) |
| Three | Large Drop | 0.88 (0.03) | 0.27 (0.03) |
|  | Medium Drop | 0.87 (0.03) | 0.17 (0.02) |
|  | Small Drop | 0.83 (0.04) | 0.13 (0.02) |

## Exploring Model Comparison Result for the Start-Low Condition in Experiment Two

To understand why the recency-based memory sampling model (with weights proportionate to the delta rule) was supported above the two-kernel delta rule for the Start-Low Condition in Experiment 2, we investigated individual-participant judgements of reliability. Figure S21 plots histograms of the reliability judgements of participants who were most consistent with the proportional memory sampling model, and Figure S22 the results of participants consistent with the two-kernel delta rule model.

**Figure S21**

*Histograms of Individual-Participant Reliability Judgements in Experiment 2, for Participants Best Fitted by the Memory-Sampling Model Where Sample Probabilities were Proportionate to Delta-Rule Weights
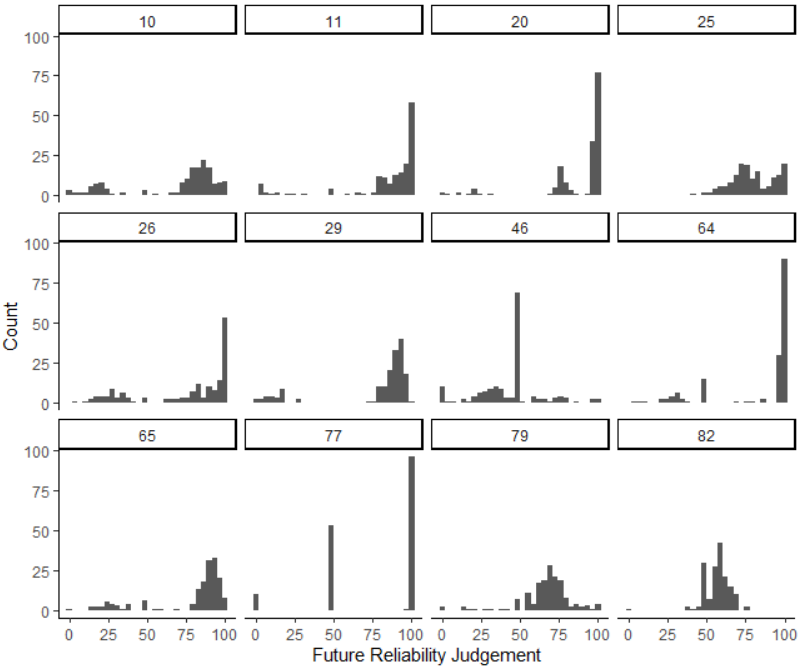
*

**Figure S22**

*Histograms of Individual-Participant Reliability Judgements in Experiment 2 for Participants Best Fitted by the Two-Kernel Delta Rule Model*


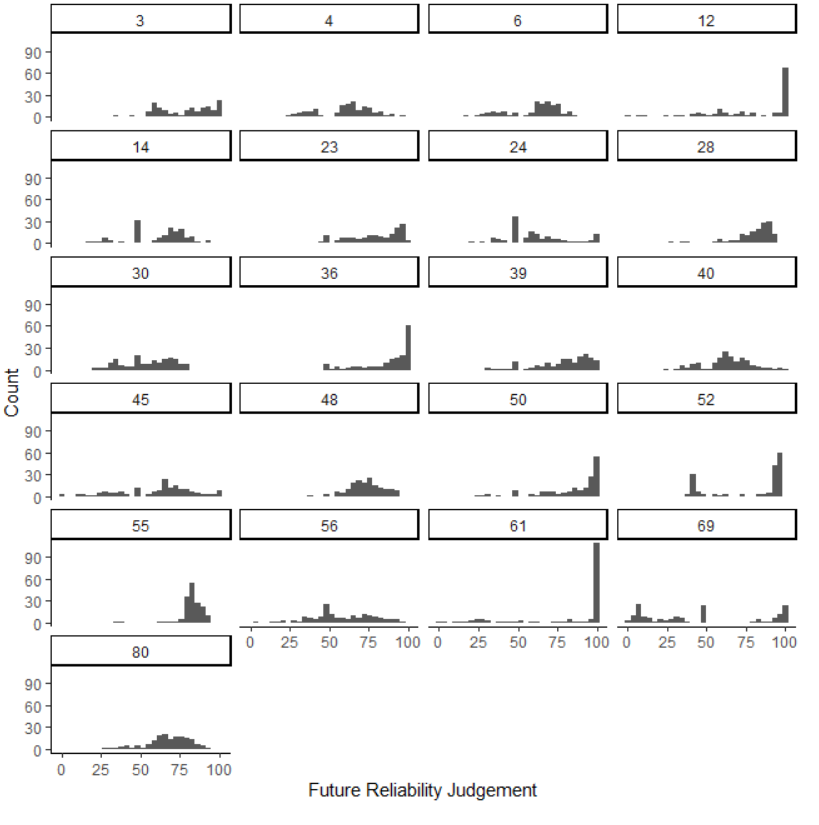


One particular participants’ reliability judgements stood out: participant 77 (Start-Low condition). This participant only made reliability judgements of 0, 50% reliable, and 100% reliable. The participant’s estimate of $r_{0}$ was very close to 50%, sampling memories of incorrect automation leads to a mean reliability estimate of 0%, and sampling memories of correct automation leads to a mean estimate of 100%. Thus, the model was able to achieve a very low log likelihood (and hence BIC) for all trial types by setting σ to a very low value. When this participant was excluded from the overall results, the two-kernel delta rule model was most favoured by BIC in the Start-Low condition, the single-process delta-rule model being second most favoured (BIC difference from two-kernel model = 179), and memory sampling according to delta-rule weights being the third most-favoured model (BIC difference from two-kernel model = 379). These results are more in line with those of the other seven between-subjects experimental conditions.

**Exploration of Why the Two-Kernel Delta-Rule Outperformed the Delta Rule Model**

Although fits of the single-process delta-rule model are reasonable, it somewhat underestimates the effect of the automation’s most recent accuracy on reliability judgements, as compared with the two-kernel delta-rule model reported in text (see Figure S16).

We suspected the fitted single-process delta-rule model did not fully fit to recency effects because such effects would require a larger learning rate, which could be inconsistent with the observed medium-term effects (i.e., applying to series of subsequent judgements) of true reliability states. To test this, we forced the single-process delta-rule model to have learning rates more consistent with the size of the effect of the most recent automation accuracy, by adding 0.1 to each participants’ rate (up to a maximum of 1), and re-simulating. As revealed in Figure S23, this allowed the delta rule model to better capture the overall magnitude of effects of the most recent automation but caused miss-fit to longer-running trends (e.g., blocks 9-13 in the Start-high condition).

**Figure S23**

*Fits of the Single-Process Delta Rule Model to Participant Reliability Judgements from Experiment 2, With Learning Rates Increased*


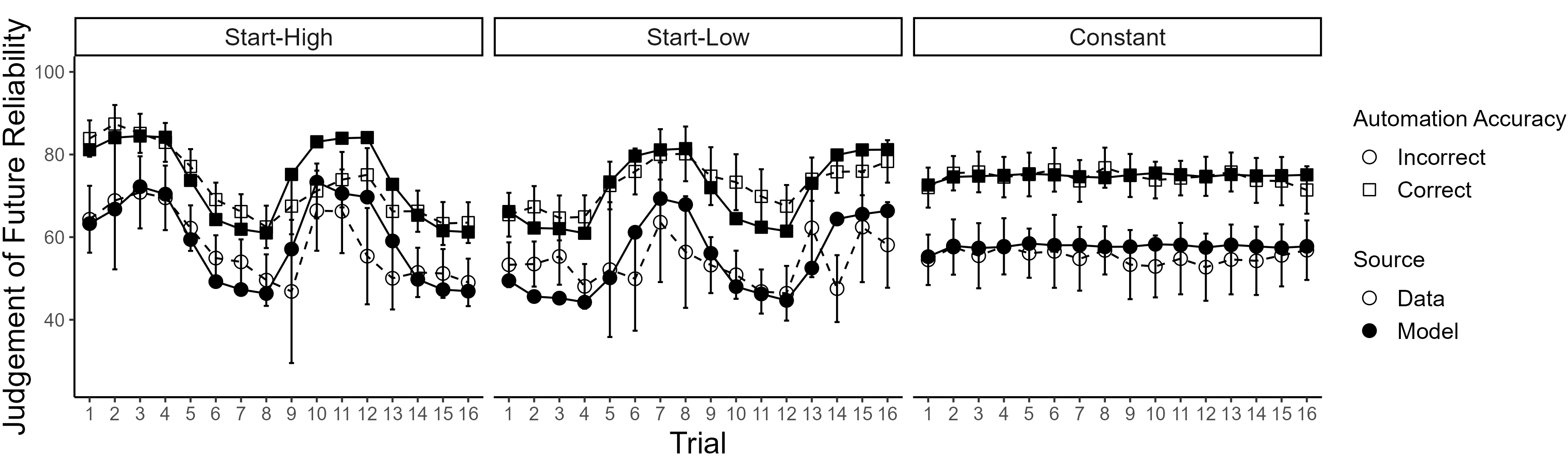


*Note:* The data corresponds to the white circles, the model mean predictions to the black dots. The error bars display the data means plus or minus the standard error.

To investigate how the two-kernel rule better fitted the combination of recent automation effects and longer-running effects of the true reliability state, we examined predictions from the model’s fast learning process and slow learning process individually. In the top panel of Figure S24, we plot predictions from the faster learning process. Unsurprisingly, these predictions are consistent with a strong effect of the most recent automation advice accuracy on reliability judgements, and in fact they predict a stronger effect than that observed. In the bottom panel of Figure S22, we plot predictions from the slow learning process. This process indicates very little effect of the most recent automation advice accuracy. Switching between these two processes allows the two-kernel model to achieve superior fits to the single-process delta rule model.

**Figure S24**

*Separate Fits of Each of the Two Kernels from the Two-Kernel Rule Model to Participant Automation Reliability Judgements in Experiment 2*


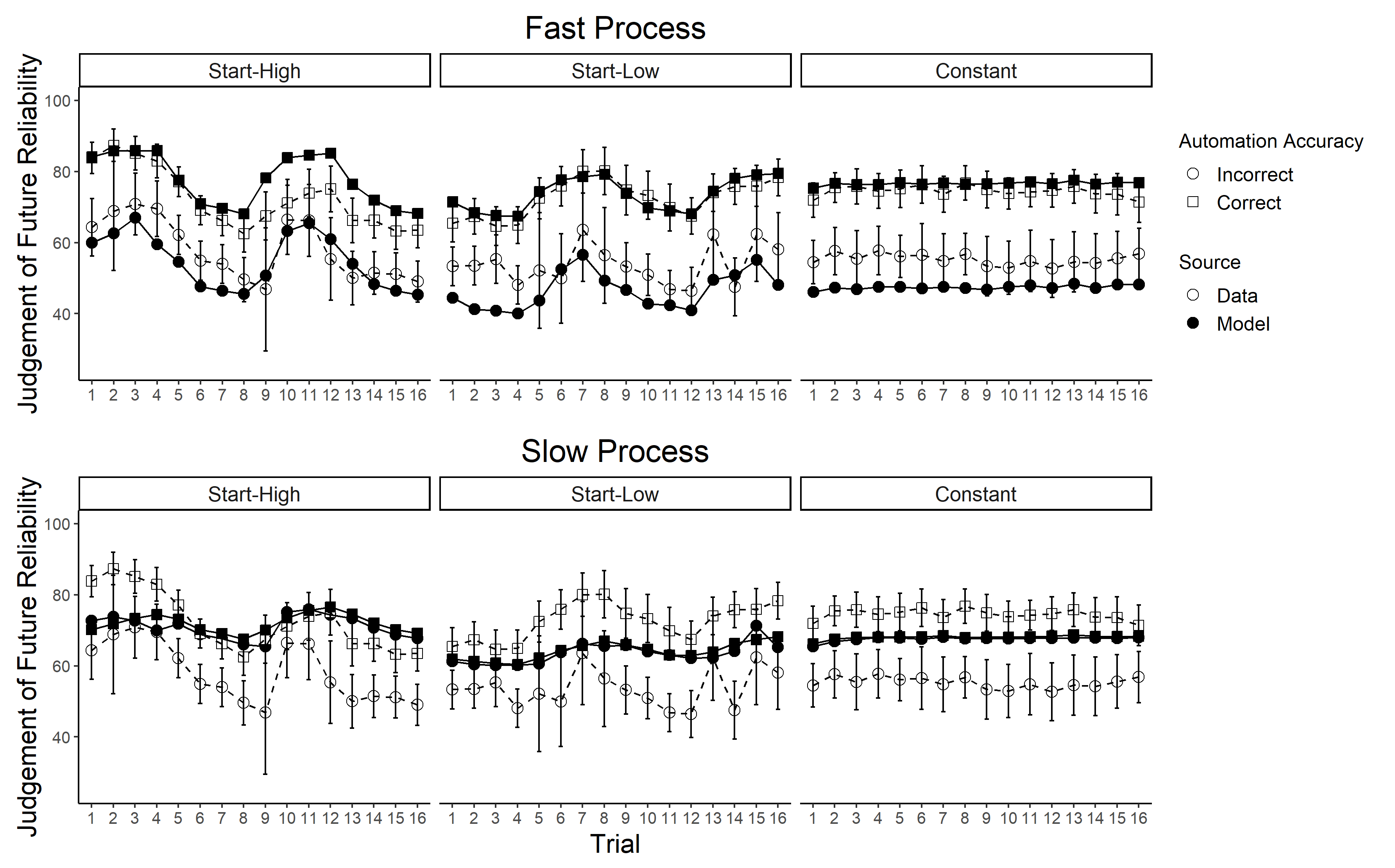


*Note.* Panel 1 contains fits of the “fast” process, and Panel 2 contains fits of the “slow” process. The data corresponds to the white circles, the model mean predictions to the black dots. The error bars display the data means plus or minus the standard error.

To understand the extent of switching between fast and slow delta learning processes, we calculated the probability of using each learning process for each participant. Figure S25 plots histograms of the probability of using the faster learning process for each participant. We observed considerable variability in the degree of switching between learning processes across participants, with a substantial number of participants at zero or near-zero switching probabilities. However, for participants for whom BIC most supported the two-kernel delta rule (Figure S26), switch rates tended to be higher than zero, and we verified that none was exactly zero (because in that case, the simpler single-process delta rule should have been preferred).

**Figure S25**

*Histograms of the Proportions of Judgements That Each Participant Made Using the “Fast” Process According to the Two-Kernel Delta Rule Model*


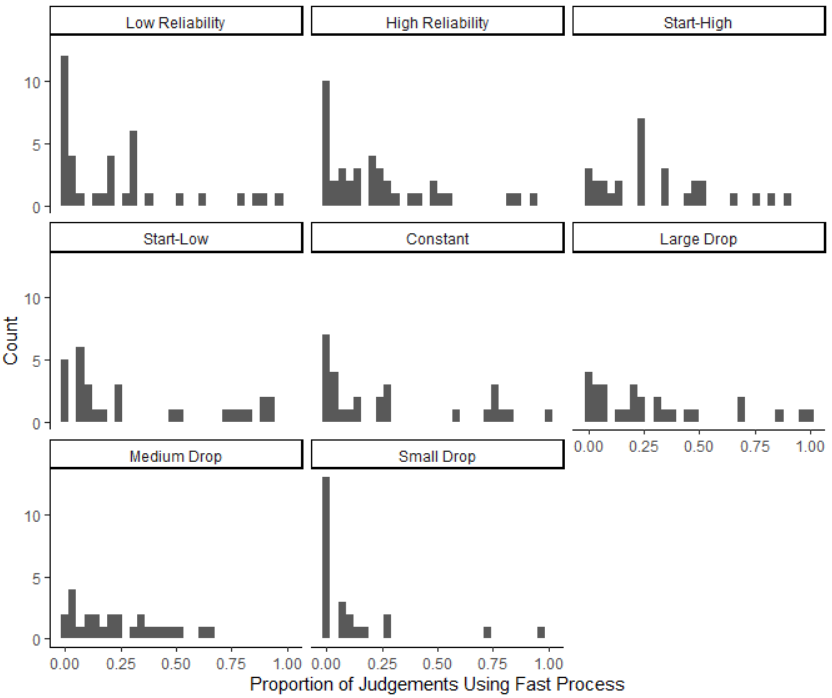


**Figure S26**

*Histograms of the Proportions of Judgements That Each Participant Made Using the “Fast” Process According to the Two-Kernel Delta Rule Model. Only for Participants Where the Two-Kernel Delta Rule was the Most Supported Model, with Substantially More Support Than the Next Best Model (BF>3.2)*


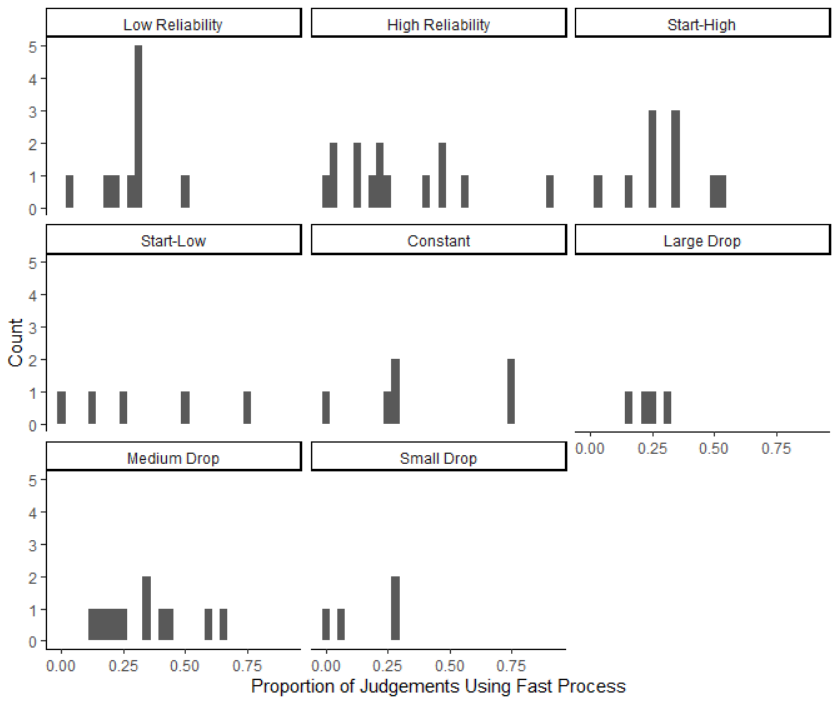


## Statistical Comparisons of Model Parameters Across Experimental Conditions

**Table S10**

*Experiment 1, Results of Independent T-Tests on Two-Kernel Delta Rule Model Parameter Values*

| Parameter Difference  (Low Reliability – High Reliability) | *df* | *t* | *p* |
| --- | --- | --- | --- |
| $r_{0}$ | 73.70 | -2.97 | .004 |
| αslow | 73.45 | 0.25 | .81 |
| αfast | 73.45 | 0.38 | .70 |
| *T* | 73.15 | 0.55 | .59 |
| σ | 71.42 | 0.24 | .81 |

**Table S11**

*Experiment 2, Results of Between-Subjects Anovas on Two-Kernel Delta Rule Model Parameter Values*

| Parameter Effect | *df_1_* | *df_2_* | *F* | *p* |
| --- | --- | --- | --- | --- |
| $r_{0}$ | 2 | 83 | 0.49 | .62 |
| αslow | 2 | 83 | 1.70 | .19 |
| αfast | 2 | 83 | 0.65 | .53 |
| *T* | 2 | 83 | 0.14 | .87 |
| σ | 2 | 83 | 2.74 | .07 |

**Table S12**

*Experiment 3, Results of Between-Subjects Anovas on Two-Kernel Delta Rule Model Parameter Values*

| Model Parameter | *df_1_* | *df_2_* | *F* | *p* |
| --- | --- | --- | --- | --- |
| $r_{0}$ | 2 | 75 | 0.72 | .49 |
| αslow | 2 | 75 | 0.83 | .44 |
| αfast | 2 | 75 | 3.06 | .052 |
| *T* | 2 | 75 | 2.77 | .07 |
| σ | 2 | 75 | 5.30 | .01 |

**Table S13**

*Experiment 3, Results of Follow-Up Independent Samples T-Tests on Differences Between Conditions in the σ Parameter*

| Parameter Difference | *df* | *t* | *p* |
| --- | --- | --- | --- |
| Large Drop – Small Drop | 49.60 | 2.11 | .04 |
| Large Drop – Medium Drop | 42.40 | 3.11 | .004 |
| Medium Drop – Small Drop | 40.83 | -0.86 | .40 |

## Exploring Bias in Probability Estimation (Linear in Log Odds Transformation)

The linear in log odds (LLO) model can be described by the below equation (Zhang & Maloney, 2012):

$\log\left( \frac{p_{est}}{1- p_{est}} \right)=\gamma\log\left( \frac{p}{1- p} \right)+ (1- \gamma)log(\frac{p_{0}}{1- p_{0}})$ (S1)

Where $p_{est}$ is the final probability estimate and $p$ is the “true” probability. The LLO transformation always outputs $p_{est}$ of 0 for $p$ of 0, and $p_{est}$ of 1 for $p$ of 1. It distorts other values according to an S- or inverse S-shaped function. The parameter $p_{0}$ identifies a point in the function at which $p_{est}$ = $p$. When $\gamma$ <1, probabilities are overestimated below $p_{0}$ and underestimated above $p_{0}$. When $\gamma$ > 1, probabilities are underestimated below $p_{0}$ and overestimated above. When $\gamma$=1, probabilities are not distorted. Figure S27 below illustrates an example where $p_{0}$ =0.4, $\gamma$ = 0.6, which approximately matches decision weights from cumulative prospect theory (Tversky & Kahneman, 1992; Zhang & Maloney, 2012).

Where possible, we augmented learning models to incorporate the LLO transformation at the production of judgements. Specifically, the output of the learning processes was treated as the “true” automation reliability ($p$ in equation S1), with the parameters $\gamma$ and $p_{0}$ estimated for each participant, so that we could solve for $p_{est}$ (the latent mean reliability judgement). To calculate likelihoods, $p_{est}$ was set as the latent mean of a truncated normal distribution with bounds between 0 and 1.

The LLO transformation could be meaningfully implemented for the two-kernel delta, delta, sampling (last/average), IIAB, Bayesian, and contingent sampling models. However, for the “no updating” model, predicted automation reliability judgements were a constant value determined by an intercept parameter (plus noise). Shifting this constant value with the LLO transformation could not achieve anything beyond shifting the intercept parameter. Similarly, the LLO transformation could not meaningfully affect predictions of the model that sampled individual memories of previous automation accuracy (using delta-rule weights). In this sampling model, a single automation experience is remembered, and thus sampled automation reliability is either 0 or 1, which are not affected by the LLO transformation. Although the model specifies that participants can also remember their estimated pre-experiment belief, *r0_sampling_recency_*, this is the only potential memory sample that could be affected by the LLO distortion. Applying the LLO function purely to *r0_sampling_recency_* could not achieve anything beyond simply shifting the value of *r0_sampling_recency_*. As the LLO transformation could not benefit either of these models, it was not applied.

**Figure S27**

*An Example of How the Linear in Log Odds Transformation Might Distort Probability Estimates*


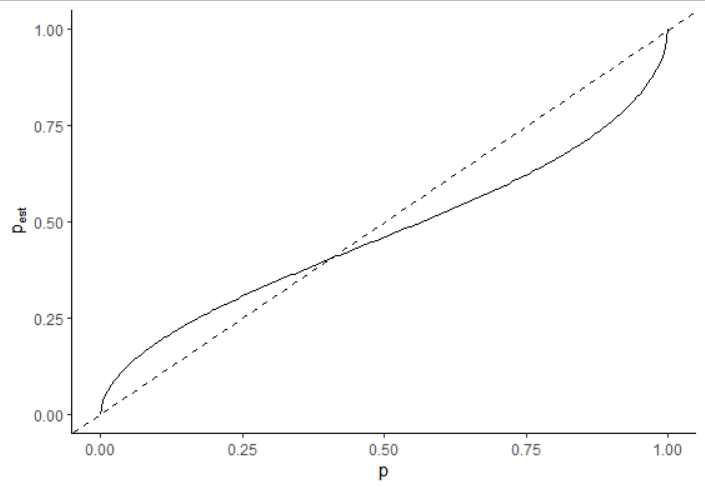


*Note.* This example, where *p_0_* =0.4 and γ = 0.6, approximately matches decision weights from cumulative prospect theory (Tversky & Kahneman, 1992; Zhang & Maloney, 2012).

Model parameters were estimated in a similar fashion to the fits presented in the main paper. The parameter bounds and start-point range are in Table S14. Almost the entire range of $p_{0}$ was possible, with bound at extreme values (>0.99 and <0.01) to avoid numerical issues. We also bounded $\gamma$ at extreme values to avoid numerical issues (see Figure S28). The lower bound, 0.1, allowed for a function that was almost a flat line (many $p_{est}$ values close to $p_{0}$), except for *p* very close to 0 (where it quickly approached 0) or 1 (where it quickly approached 1). The upper bound, 5, allowed for a low $p_{est}$ values (near 0) up until $p$ = $p_{0}$, with $p_{est}$ then quickly climbing to values near 1. Due to the non-linear effects of $\gamma$_,_ we estimated it on the log scale.

**Table S14**

*The parameter bounds and ranges of start points that were sampled for parameters relevant to the “linear in log odds” transformation (Zhang & Maloney, 2012). We tried hand-picked start points (rightmost column), as well as sampling start points from a grid (constrained by the ranges in the second rightmost column)*

| Parameter | Bounds | Start point range | Hand-picked start point |
| --- | --- | --- | --- |
| $p_{0}$ | 0.01-0.99 | 0.02-0.98 | 0.5 |
| *log(γ)* | log(0.1)-log(5) | log(0.11)-log(4.99) | log(1) |

**Figure S28**

*An Illustration of the Parameter Bounds on γ Affecting the LLO Function*


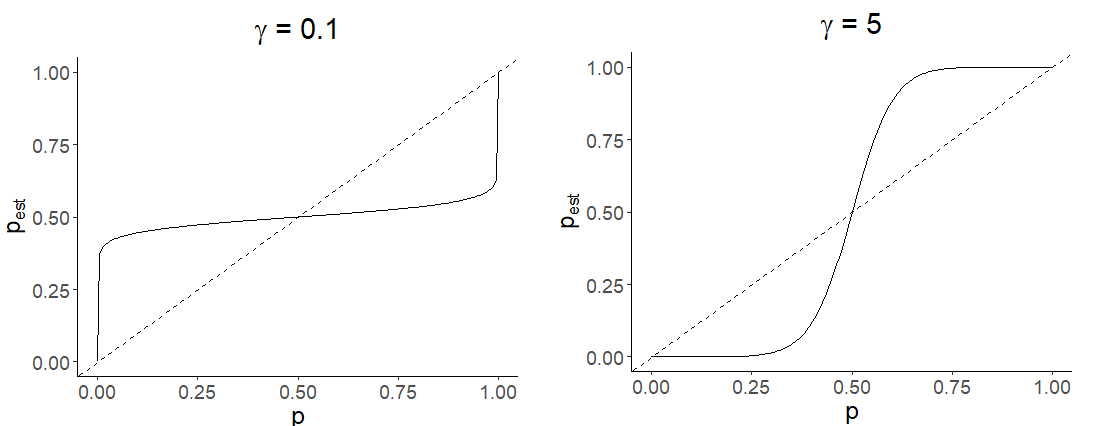
*Note:* For both plots, the crossover point $p_{0}$ was set to 0.5*.*

In Table S15, we summarise group BIC model comparison results regarding our LLO augmented models. For the two aforementioned models where the LLO transformation could have no benefit, the same model fits were used as reported in text. Overall, our results are consistent with those reported in text in supporting the two-kernel delta rule and delta rule models. The two-kernel delta rule model is the most favoured for every experimental condition. This is a slight improvement in support over the results reported in the main text, where an unusual pattern of responding from a single participant results drove a memory sampling model ahead of the two-kernel delta rule in the “Start-Low” condition. Fits of the augmented two-kernel delta rule model to participant data look similar to the fits reported in the original model in text (Figure S29). The delta-rule model was the second most supported in all conditions except the Start-Low condition. Rank ordering of the other alternative models is similar to in text, although not identical. Models augmented with the LLO transformation generally benefitted relative to the two models that were not.

**Table S15**

*Group BIC values (summed across participants) for each model for each experimental condition. In this supplementary analysis, models were augmented with the “linear in log odds”(LLO) transformation (Zhang & Maloney, 2012) on their final reliability judgements. Two models could not be augmented, due to the nature of their prediction: the no updating model, and the sampling model that sampled individual memories of experience (proportional to delta-rule weights). We report BIC values after subtracting the BIC for the most supported model for each experiment condition (hence, the most supported model for each experimental condition has a value of 0). We report BIC in this manner because it is the differences between BICs that matter for the purposes of model comparison (Kass & Raftery, 1995) and it is easier to see which model is best fitting for each condition, and the relative performance of other models to that best fitting model.*

|  |  |  | Experiment | | |  |  |  |
| --- | --- | --- | --- | --- | --- | --- | --- | --- |
|  | One | | Two | | | Three | | |
| Model | High | Low | Start-High | Start-Low | Constant | Large Drop | Medium Drop | Small Drop |
| Two-kernel Delta | 0 | 0 | 0 | 0 | 0 | 0 | 0 | 0 |
| Delta | 245 | 205 | 623 | 264 | 501 | 38 | 102 | 166 |
| Sampling (proportional to delta weights) | 2850 | 2598 | 915 | 2850 | 2598 | 2850 | 2598 | 915 |
| Sampling (last/average) | 889 | 1128 | 1728 | 950 | 849 | 2287 | 1545 | 302 |
| IIAB | 1893 | 1607 | 1493 | 1377 | 2217 | 1278 | 1091 | 692 |
| Bayesian | 1227 | 1207 | 2119 | 1976 | 1786 | 3292 | 1713 | 527 |
| Contingent Sampling | 3782 | 3699 | 3927 | 3098 | 4268 | 2943 | 2872 | 2049 |
| No updating | 2129 | 2011 | 4466 | 2264 | 2208 | 4279 | 2748 | 1092 |
|  |  |  |  |  |  |  |  |  |

**Figure S29**

*Averaged Predictions of the Two Kernel Delta-Rule Model*


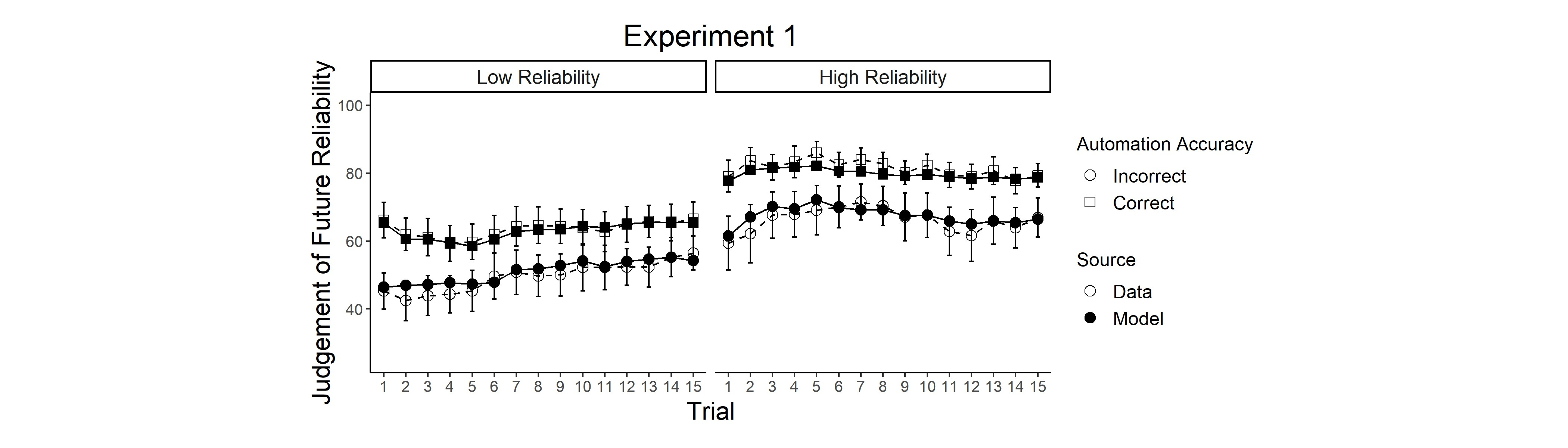


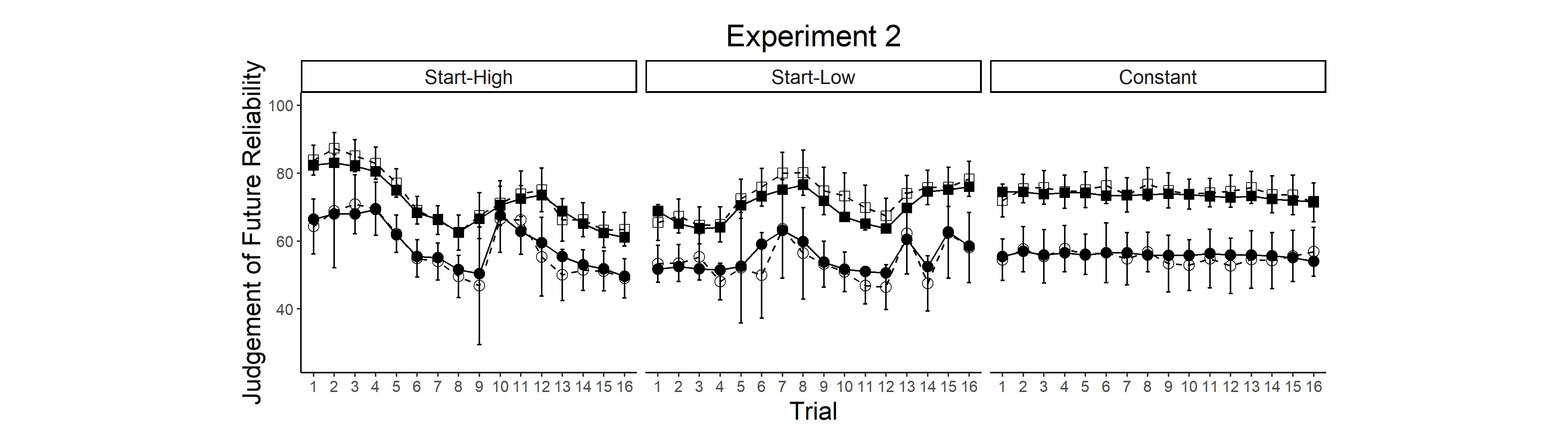


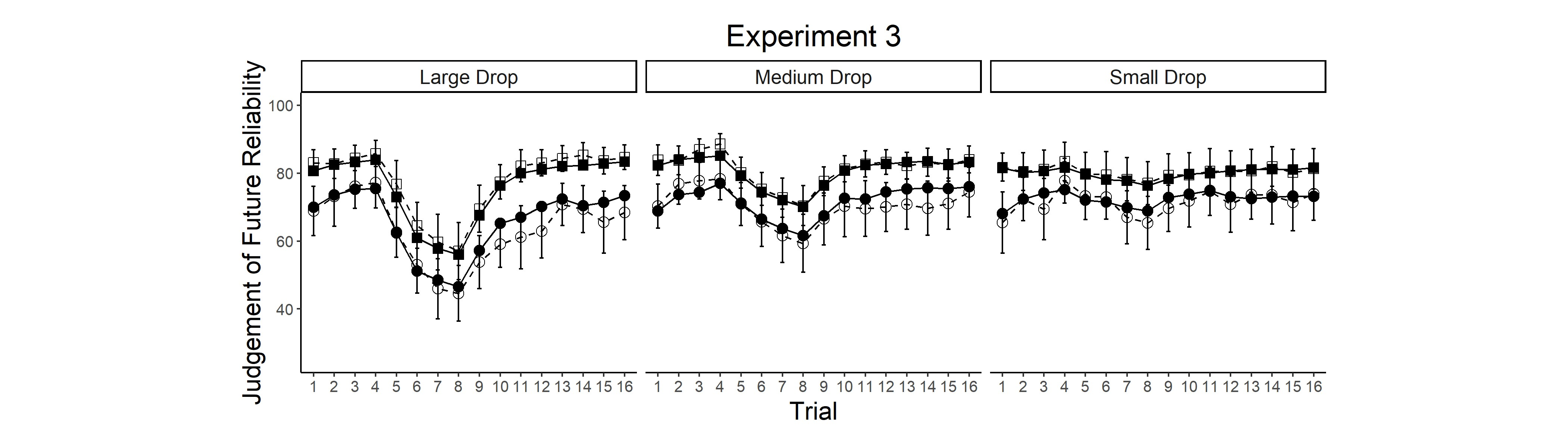


*Note.* In this supplementary analysis, the model was augmented with the “linear in log odds” transformation (Zhang & Maloney, 2012), which affected final reliability judgements.

In Table S16, we present results relating to LLO augmented model heterogeneity across participants. Similar to the modelling in the main text, approximate Bayes Factors indicated conclusive results for 83% of participants. The percentages that follow refer to results within that subset. As with the reports resulted in main text, the two-kernel delta rule and delta rule models were the most commonly supported. Interestingly, support for these models was more consistent than in text, now best accounting for 58%-84% of participants, depending on condition. However, there was still heterogeneity across participants. Recency-based memory sampling models were still somewhat represented, accounting for 0-29% of total participants, depending on condition. The IIAB model was less supported than in text, accounting for only 0-7% of participants. The Bayesian model was slightly more supported than in text, accounting for 0-13% of participants. The contingent sampling model accounted for 0-4% of participants. Notably, the no updating model (which had no LLO augmentation) was less strongly represented than in text, accounting for 0-14% of participants.

**Table S16**

*The Proportion of Participants for Whom BIC Conclusively Favoured Each Model for Each Experimental Condition. Presented as a Percentage Of the 83% Of Participants for Which One Model was Conclusively Favoured. In This Supplementary Analysis, Models Were Augmented with the “Linear In Log Odds” Transformation (Zhang & Maloney, 2012) On Their Final Reliability Judgements. Two Models Could Not Be Augmented, Due to the Nature Of Their Prediction: The No Updating Model, and the Sampling Model That Sampled Individual Memories Of Experience (Proportional to Delta-Rule Weights).*

|  |  |  | Experiment | | |  |  |  |
| --- | --- | --- | --- | --- | --- | --- | --- | --- |
|  | One | | Two | | | Three | | |
| Model | High | Low | Start-High | Start-Low | Constant | Large Drop | Medium Drop | Small Drop |
| Two-kernel Delta | 53% | 50% | 46% | 40% | 50% | 17% | 33% | 43% |
| Delta | 13% | 27% | 38% | 20% | 8% | 43% | 43% | 29% |
| Sampling (proportional to delta weights) | 7% | 0% | 0% | 20% | 17% | 4% | 0% | 0% |
| Sampling (last/average) | 10% | 0% | 12% | 4% | 12% | 13% | 5% | 10% |
| IIAB | 0% | 7% | 0% | 4% | 4% | 4% | 5% | 5% |
| Bayesian | 13% | 7% | 0% | 0% | 4% | 4% | 0% | 10% |
| Contingent Sampling | 3% | 0% | 0% | 0% | 4% | 4% | 0% | 0% |
| No updating | 0% | 10% | 4% | 12% | 0% | 9% | 14% | 5% |
|  |  |  |  |  |  |  |  |  |

Issues with LLO parameter estimation. When combining the LLO transformation with learning models, one concern is the possibility of excessive model flexibility, relative to the available constraint in the data. This can cause issues both with model comparison and parameter estimation. Although our LLO model fits ultimately converged, some of our results point to possible issues with combined estimation of parameters of the learning processes and LLO transformation, given our available data.

Figure S30 demonstrates an example of the substantial variability in $\gamma$ for the augmented two-kernel delta rule model. We found that estimates of $\gamma$ varied widely, with some participants lower probabilities are underestimated ($\gamma$ < 1), and others suggesting lower probabilities are overestimated ($\gamma$ > 1). This variability suggests that biases in automation reliability estimation were not consistent across participants. Further, pathological LLO estimation results were evident in some cases. This is well-illustrated by the contingent sampling model. For this model, many estimates of $\gamma$ were very close to the parameter bounds of 0.1 and 5 (see Figure S31 below). These bounds imply relatively implausible probability distortions, and thus a tendency for parameters to consistently hit the bounds may imply overfitting.

Overall, although the LLO-augmented model analysis converged with our conclusions in text, we decided to focus on the former because 1) the LLO transformation could not be applied to all the learning models considered, 2) the transformation did not suggest consistency in biases across participants and 3) we had concerns about model flexibility and parameter estimation.

## Figure S30

## *Histogram of Individual-Participant Estimates of* $\boldsymbol{\gamma}$ *from the Augmented Two-Kernel Delta Rule Model, High Reliability Condition of Experiment One.*


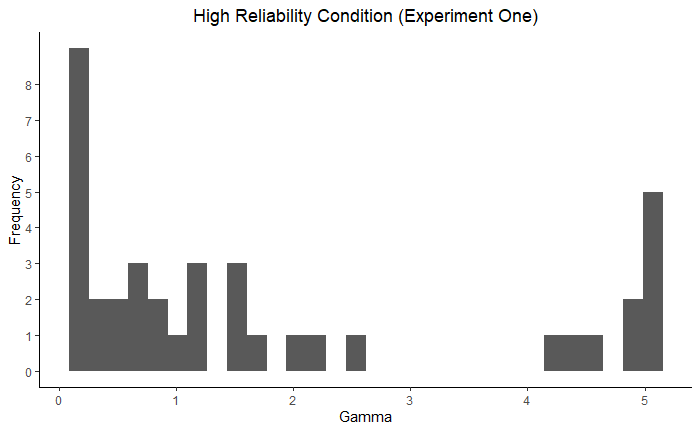


## Figure S31

## *Histogram of Individual-Participant Estimates of* $\boldsymbol{\gamma}$ *from the Augmented Contingent Sampling Model, High Reliability Condition of Experiment One*
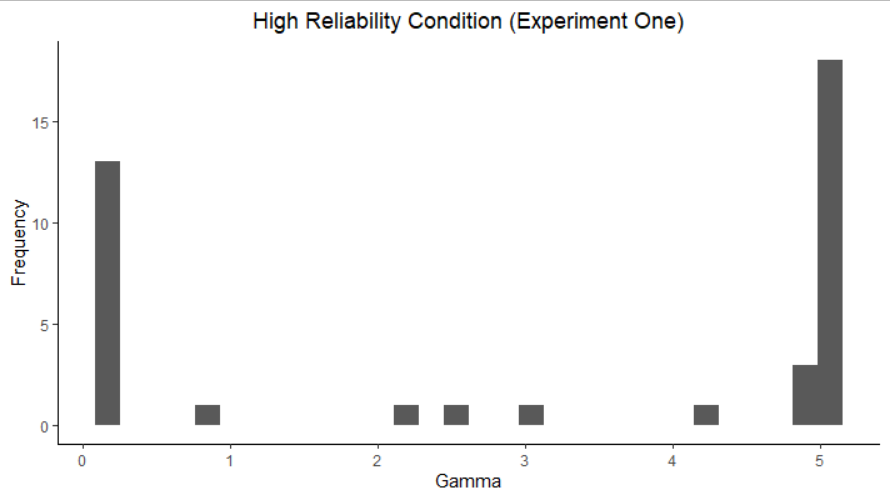


# References

Gallistel, C. R., Krishan, M., Liu, Y., Miller, R., & Latham, P. E. (2014). The perception of probability. Psychological Review, 121(1), 96–123. https://doi.org/10.1037/a0035232

Hutchinson, J., Strickland, L., Farrell, S., & Loft, S. (2022a). Human behavioral response to fluctuating automation reliability. Applied Ergonomics, 105, 103835. https://doi.org/10.1016/j.apergo.2022.103835

Hutchinson, J., Strickland, L., Farrell, S., & Loft, S. (2022b). The perception of automation reliability and acceptance of automated Adviceadvice. Human Factors, 00187208211062985. https://doi.org/10.1177/00187208211062985

Varadhan, R., Borchers, H. W., & Bechard, V. (2020). *_dfoptim: Derivative-Free Optimization_. R package version 2020.10-1*. https://CRAN.R-project.org/package=dfoptim

Zhang, H., & Maloney, L. T. (2012). Ubiquitous log odds: a common representation of probability and frequency distortion in perception, action, and cognition*. Frontiers in Neuroscience, 6*, 1. https://doi.org/10.3389/fnins.2012.00001
